# Clinical evaluation of smartphone-based fluorescence imaging for guidance and monitoring of ALA-PDT treatment of early oral cancer

**DOI:** 10.1117/1.JBO.25.6.063813

**Published:** 2020-04-11

**Authors:** Shakir Khan, M. A. Bilal Hussain, Amjad P. Khan, Hui Liu, Shaista Siddiqui, Srivalleesha Mallidi, Paola Leon, Liam Daly, Grant Rudd, Filip Cuckov, Colin Hopper, Stephen G. Bown, Kafil Akhtar, Syed Abrar Hasan, Shahid Ali Siddiqui, Tayyaba Hasan, Jonathan P. Celli

**Affiliations:** a Aligarh Muslim University, Jawaharlal Nehru Medical College, Department of Radiotherapy, Aligarh, India; b Massachusetts General Hospital and Harvard Medical School, Boston, Massachusetts, United States; c University of Massachusetts at Boston, Boston, Massachusetts, United States; d Aligarh Muslim University, Jawaharlal Nehru Medical College, Department of Radiodiagnosis, Aligarh, India; e University College London, London, England, United Kingdom; f Aligarh Muslim University, Jawaharlal Nehru Medical College, Department of Pathology, Aligarh, India; g Aligarh Muslim University, Jawaharlal Nehru Medical College, Department of Otorhinolaryngology (E.N.T.), Aligarh, India

**Keywords:** oral cancers, smartphone, photodynamic therapy, protoporphyrin IX, fluorescence imaging

## Abstract

**Significance:** India has one of the highest rates of oral cancer incidence in the world, accounting for 30% of reported cancers. In rural areas, a lack of adequate medical infrastructure contributes to unchecked disease progression and dismal mortality rates. Photodynamic therapy (PDT) has emerged as an effective modality with potential for treating early stage disease in resource-limited settings, while photosensitizer fluorescence can be leveraged for treatment guidance.

**Aim:** Our aim was to assess the capability of a simple smartphone-based device for imaging 5-aminolevulinic acid (ALA)-induced protoporphyrin IX (PpIX) fluorescence for treatment guidance and monitoring as part of an ongoing clinical study evaluating low-cost technology for ALA-based PDT treatment of early oral cancer.

**Approach:** A total of 29 subjects with <2  cm diameter moderately/well-differentiated microinvasive (<5  mm depth) oral squamous cell carcinoma lesions (33 lesions total, mean area $∼1.23\;{\mathrm{cm}}^{2}$) were administered 60  mg/kg ALA in oral solution and imaged before and after delivery of $100\;{J/\mathrm{cm}}^{2}$ total light dose to the lesion surface. Smartphone-based fluorescence and white light (WL) images were analyzed and compared with ultrasound (US) imaging of the same lesions.

**Results:** We present a comparative analysis of pre- and post-treatment fluorescence, WL, and US images of oral lesions. There was no significant difference in the distribution of lesion widths measured by fluorescence and US (mean widths of 14.5 and 15.3 mm, respectively) and linear regression shows good agreement ($R^{2}=0.91$). In general, PpIX fluorescence images obtained prior to therapeutic light delivery are able to resolve lesion margins while dramatic photobleaching (∼42%) is visible post-treatment. Segmentation of the photobleached area confirms the boundaries of the irradiated zone.

**Conclusions**: A simple smartphone-based approach for imaging oral lesions is shown to agree in most cases with US, suggesting that this approach may be a useful tool to aid in PDT treatment guidance and monitoring photobleaching as part of a low-cost platform for intraoral PDT.

## 1 Introduction

The increasing incidence of head and neck cancers in South Asia has been described as a global health crisis.^1^ Particularly in India, the high incidence of oral cancers is ascribed to the popularity of chewing “gutka” (a compound mixture of tobacco, acacia, and betel nut extracts). The problem of high oral cancer incidence is exacerbated, particularly in rural areas, by limited accessibility of early stage medical screening and imaging. Furthermore, the economic burden of late stage treatments such as complex surgical procedures and/or radiation therapy pose a further barrier in oral cancer management.^2^ Photodynamic therapy (PDT) has emerged as an alternative and noninvasive early stage anticancer treatment modality.^3^ PDT is a treatment in which a precursor or photosensitizer drug localized to the lesion sites is activated by light to generate singlet oxygen (O21)-mediated photocytotoxicity against cancer cells. Here, we used 5-aminolevulinic acid (ALA as Levulan^®^, DUSA, SUN Pharmaceuticals, Inc.), a precursor for the photoactive derivative protoporphyrin IX (PpIX).^4^ PpIX not only acts as a fluorescent probe but also imparts antitumor toxicity when activated by light. This dual functionality has been used successfully for image-guided treatment of various cancers, including those of the oral cavity.^5^^,^^6^ In clinical settings, PpIX fluorescence and photobleaching make it effective as a diagnostic as well as a treatment monitoring tool.^7^^,^^8^ Recently, a smartphone with fluorescence imaging capability has been used as a low-cost device for premalignant oral screening.^8^ The wide availability and popularity of smartphones, particularly in the developing countries, make this device promising as a low-cost, portable, and capable theragnostic cancer technology for global health.^9^^–^^11^ Building on previous preliminary findings,^12^ we report here an evaluation of this simple and low-cost fluorescence imaging approach for guidance and monitoring of PDT treatment in the clinic.

## 2 Materials and Methods

### 2.1 Subject Selection

A total of 29 subjects (4 females, 25 males, median age: 40 years, age range: 24 to 64 years) with a histologically proven $T_{1}N_{0}M_{0}$ stage oral buccal mucosa lesion (<2  cm. diameter) were enrolled in the study. Subjects were excluded if they had a history of photosensitivity or photosensitive diseases, were taking any photosensitive medications, or had any history of malignant disease treatment and had any allergies to the ALA formulation. Subjects were given written, audio, and video information on the trial and asked to give written consent prior to their participation. This study protocol was approved by the India Council of Medical Research.

### 2.2 Photodynamic Therapy Treatment, Diagnostic, and Monitoring Timeline

**Fig. 1:**
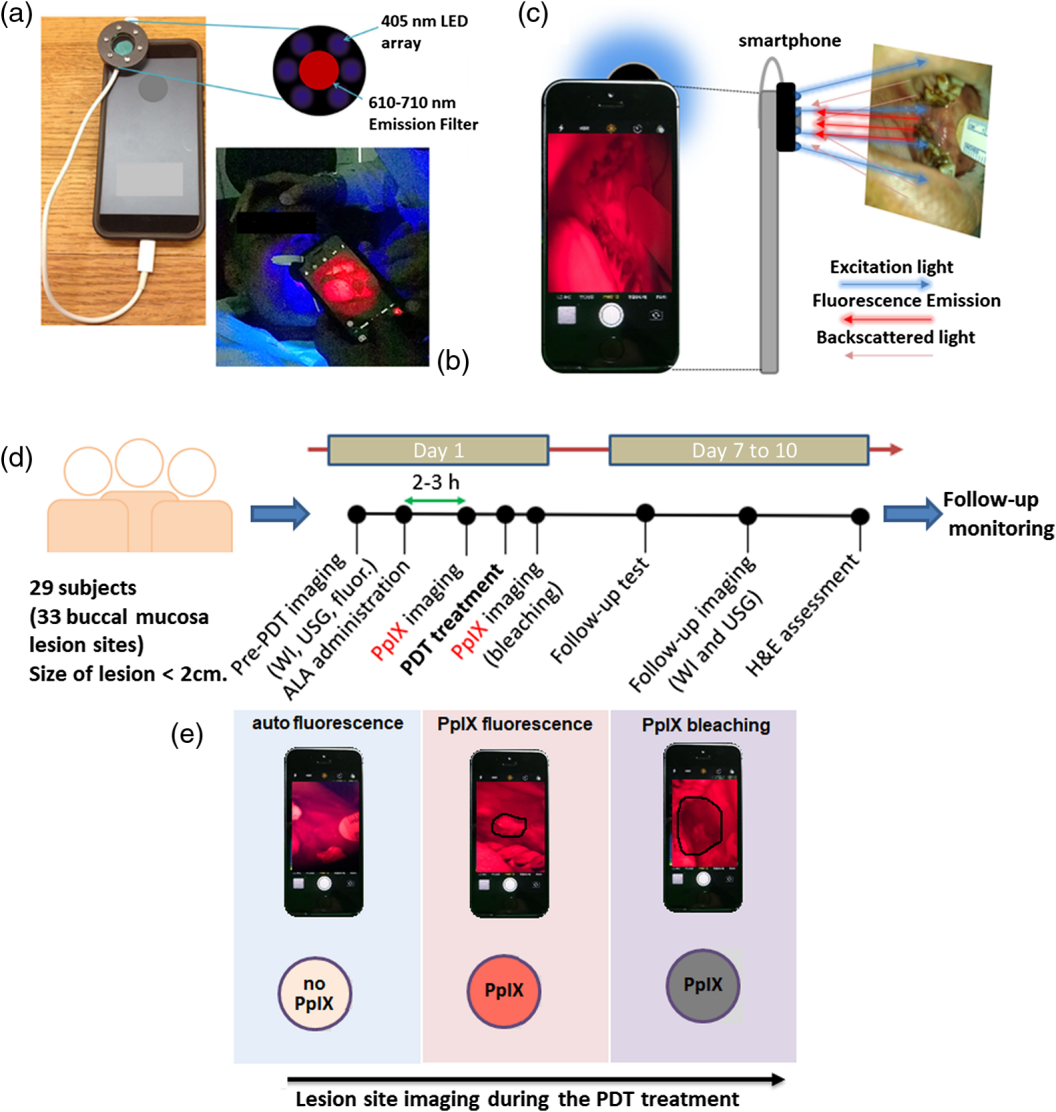
Smartphone device and workflow for PpIX fluorescence and WL imaging. (a) A smartphone attached with a 405-nm LED array (modified FluoroVu device, by Eigen Imaging) fitted with a 610- to 710-nm emission filter.^17^ (b) Handheld smartphone-based lesion site PpIX fluorescence imaging during the buccal mucosa PDT treatment. (c) The methodology of fluorescence image formation using blue-violet excitation (405 nm peak; emitted from the LED array). (d) Subject treatment timeline with the pre-PDT, post-PDT, and follow-up diagnostics (i.e., WL, US, PpIX imaging, and H&E) and clinical monitoring assessments. (e) Stepwise illustrative presentation of smartphone-based lesion site PpIX fluorescence detection and after-light or post-PDT treatment bleaching.

A total of 60  mg/kg dose of 5-ALA was administered orally in three fractions (20  mg/kg each) at hourly intervals based on the regimen described by Regula et al.^13^ Light delivery started 15 min after the third dose. Light-emitting diode (LED) light (∼640  nm peak) was delivered to the target lesion using a flexible optical fiber attached to a portable LED light source, which was previously described for this application,^14^ at an irradiance of $∼50\;{\mathrm{mW}/\mathrm{cm}}^{2}$. The total light dose was $100\;{J/\mathrm{cm}}^{2}$, fractionated into 10-min periods of light delivery with 2-min breaks, proceeding until the total light dose had been delivered (approximately three fractions total). In this case, the rationale for including 2-min intervals between fractions was motivated in part by patient comfort, allowing patients a brief break before repositioning the light applicator. However, this fractionation regimen is also supported by previous work showing that reaccumulation of depleted tissue oxygen and PpIX relocalization during fractionation breaks of comparable duration contribute to improved tumor response.^15^ Light delivery to target lesions was achieved using custom light delivery applicators on the end of the delivery fiber to control the spot size and position depending upon the size of the lesion and how well the patients could open their mouth.^16^ The LED light spot covered the lesion as well as a margin of normal tissue. Before and after light delivery, the lesion was monitored by WL, ultrasound (US), PpIX fluorescence imaging, and histopathological examination using hematoxalin and eosin (H &E) stained biopsy sections. The lesion site clinical evaluations were performed during monthly follow-ups [Fig. 1(d)].

### 2.3 Smartphone-Based Protoporphyrin IX Fluorescence Imaging

Imaging of PpIX fluorescence prior to the administration of ALA, then before and after PDT was performed on 33 lesion sites on the buccal mucosa (29 patients are included in this dataset, four of whom had two lesion sites). Images were obtained using a consumer smartphone (iPhone 7, Apple Inc., Cupertino, California, iOS version 10.1, upgraded to 11.1 and 12.1 over the course of study) with a circular array of 405-nm LEDs for fluorescence excitation (modified FluoroVu device, by Eigen Imaging) mounted around the phone camera sensor fitted with a 610- to 710-nm emission filter, as previously described^17^ [Fig. 1(a)]. In sequential images of the same site (pre-ALA, then pre-PDT and post-PDT) effort was made to hold the smartphone at the same angle and distance from the target lesion in the oral cavity [Fig. 1(b)]. Backscattered blue/violet excitation light is filtered by the emission filter so that the smartphone camera captures fluorescence contrast generated by locally higher PpIX accumulation in the malignant tissue [Fig. 1(c)].

### 2.4 Multimodality Lesion-Site Imaging and Image Analysis

Prior to ALA administration, WL and autofluorescence images for each lesion were obtained using the smartphone device. The PpIX fluorescence image was taken immediately after the third dose of ALA. The post-PDT PpIX bleaching image was captured after the last fractionated light dose [Fig. 1(e)]. The US scanning was used as an independent method for validation of lesion dimensions obtained by WL and fluorescence imaging using the smartphone. The US imaging was taken before ALA administration as well as after the PDT treatment (on 7th to 10th day after PDT) [Fig. 1(d)]. Image analysis was performed on a computer using raw image data from the smartphone. Fluorescence image analysis used only the red channel, isolated from each RGB image. All image analysis was performed using NIH ImageJ software and open source Python routines.^18^^,^^19^ For the PpIX fluorescence and bleached image segmentation, each RGB image was split to a red-channel 8-bit grayscale image by ImageJ.^18^ The red-channel gray images were displayed using a 16-color lookup table (16LUT) for improved visual contrast and identification of fluorescence and bleaching at the lesion site and to help guide thresholding. Images were thresholded manually with typical threshold values for segmentation of the pre-PDT tumor area and the photobleached area of 214 and 159, respectively. The fluorescence intensity distribution was plotted using the three-dimensional (3-D) surface plot plugin function in ImageJ. The Python OpenCV package was used for fluorescence as well as WL hue, saturation, and value (HSV) image segmentation.^19^ In the WL HSV color spacing segmentation, we masked the WL original image and WL gray image with the help of tunable and threshold ranges of HSV values trackbar in the python OpenCV package.

### 2.5 Statistical Analysis

The significant and central values of PpIX fluorescence, bleaching, WL lesion, US lesion, HSV lesion, and 16LUT lesion parameters were analyzed by open source statistical software R (Comprehensive R Archive Network).^20^ The difference between mean/central values was assessed by Student’s paired t-test. The analysis of prediction capability of predictor/independent variables (i.e., lesion site PpIX fluorescence and HSV) to predict the dependent variables (i.e., US variables and 16LUT) were determined by linear regression analysis. The outliers in the linear regression analysis were identified by influence plot analysis using the “car” package in R console. Statistical analysis was performed using the “ggplot2” package in R console.

## 3 Results and Discussion

### 3.1 Protoporphyrin IX Fluorescence Imaging of Oral Lesions

**Fig. 2:**
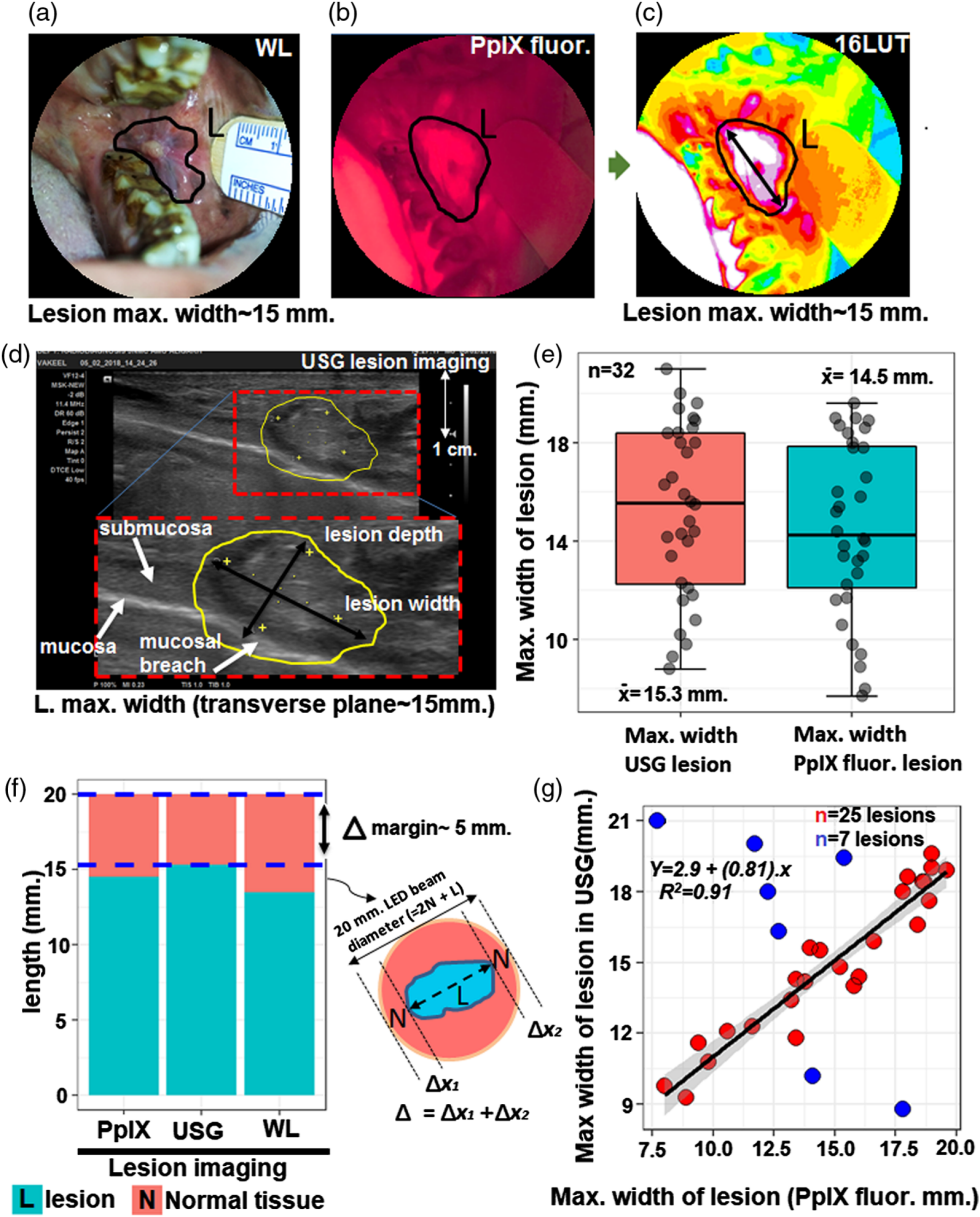
The PpIX fluorescence-based imaging of oral lesions. (a) and (b) The measurement of maximum dimension of lesion with the help of smartphone WL and fluorescence imaging. (c) The fluorescence image applied 16LUT for the measurement of maximum lesion width with visible margins. (d) US for the maximum width of the lesion in transverse plane. (e) The boxplot of the maximum lesion width measured from the US and PpIX fluorescence imaging on 32 lesion sites. (f) The barplot of length [i.e., 20 mm beam light covered area = lesion + normal tissue margins (Δ)] where maximum lesion width is measured by US, LUT, and WL imaging. (g) The linear regression graph between the PpIX fluorescence (as LUT) and US lesion width parameters. The blue dots represent the outliers superimposed in the graph. The shaded gray area represents the confidence interval (95%) for regression coefficients.

The appropriate determination of the maximum lateral extent of the lesion is critical to ensure that the beam spot fully covers the lesion and appropriate margins. Here, the maximum width of the lesion is measured by WL, PpIX fluorescence imaging, and US [Figs. 2(a), 2(b), and 2(d)]. To enhance display contrast and aid in visualization of lesion boundaries, the red channel from RGB images of PpIX fluorescence can be displayed using a 16LUT, as shown in Fig. 2(c). The US was used as an independent modality for comparison with PpIX fluorescence images [Fig. 2(d)]. The boxplot distribution of lesion width measured by US and fluorescence imaging showed nearly equal interquartile ranges with a mean 15.3 and 14.5 mm, respectively. Moreover, the averages of the lesion widths measured by US and PpIX fluorescence modalities are not significantly different (t=1.19; p=0.24) [Fig. 2(e)]. By design, this study included only $T_{1}N_{0}M_{0}$ stage oral buccal mucosa lesions (i.e., ≤20  mm diameter) and mean width of the normal tissue (i.e., margin of normal tissue=20  mm−max.lesion width) were 4.73 and 5.50 mm along the diameter in US and fluorescence imaging, respectively [Fig. 2(f)]. In addition to boxplot distributions showing nearly identical and uniform distribution of measurements in US and PpIX fluorescence, linear regression analysis was performed to examine linearity. This analysis shows that, for the majority of lesions imaged (25/32), there is a strong correlation between the maximum lesion width assessed by US and fluorescence ($R^{2}=0.91$, among 25 lesions) and, overall, within this dataset, PpIX fluorescence lesion size reliably predicts US lesion size ($p=1.8\times 10^{-13}$) [Fig. 2(g)]. The outliers in the linear regression comparison may be attributed in part to challenges in interpreting the boundaries of small intraoral lesions with US, which itself is a highly user-dependent modality with sensitivity and specificity ranging from ∼75% to 100%.^21^ Previous studies have reported that the boundaries of $T_{1}N_{0}M_{0}$ oral lesions may not be clearly discerned from the surrounding healthy tissue using US alone, unless combined with Doppler imaging or high-frequency US imaging.^22^^–^^24^ Ultimately the combination of US with surface fluorescence imaging may provide a better metric for estimating the lesion size than either modality alone.

Fluorescence background visible in surrounding normal tissue [Fig. 2(b)] is likely due to a combination of nonspecific PpIX accumulation in the oral mucosa as well as contribution from autofluorescence with overlapping spectral properties. In particular, patients in this study were clinically observed to have poor oral hygiene, leading to a potentially high fluorescence background from endogenous porphyrins found in bacteria, which has previously been implicated as a source of background for PpIX imaging in the oral cavity.^25^ In future studies, this background may be mitigated by systematic attention to oral hygiene immediately prior to fluorescence imaging. Our analysis also contains quantitative information about the shape of lesions. The 25 lesions that exhibited strong correlation between fluorescence and US image data were predominantly elongated in shape, though two were nearly circular (Fig. S1 in the Supplementary Material).

### 3.2 Lesion Site Fluorescence Analysis Pre- and Post-Photodynamic Therapy

**Fig. 3:**
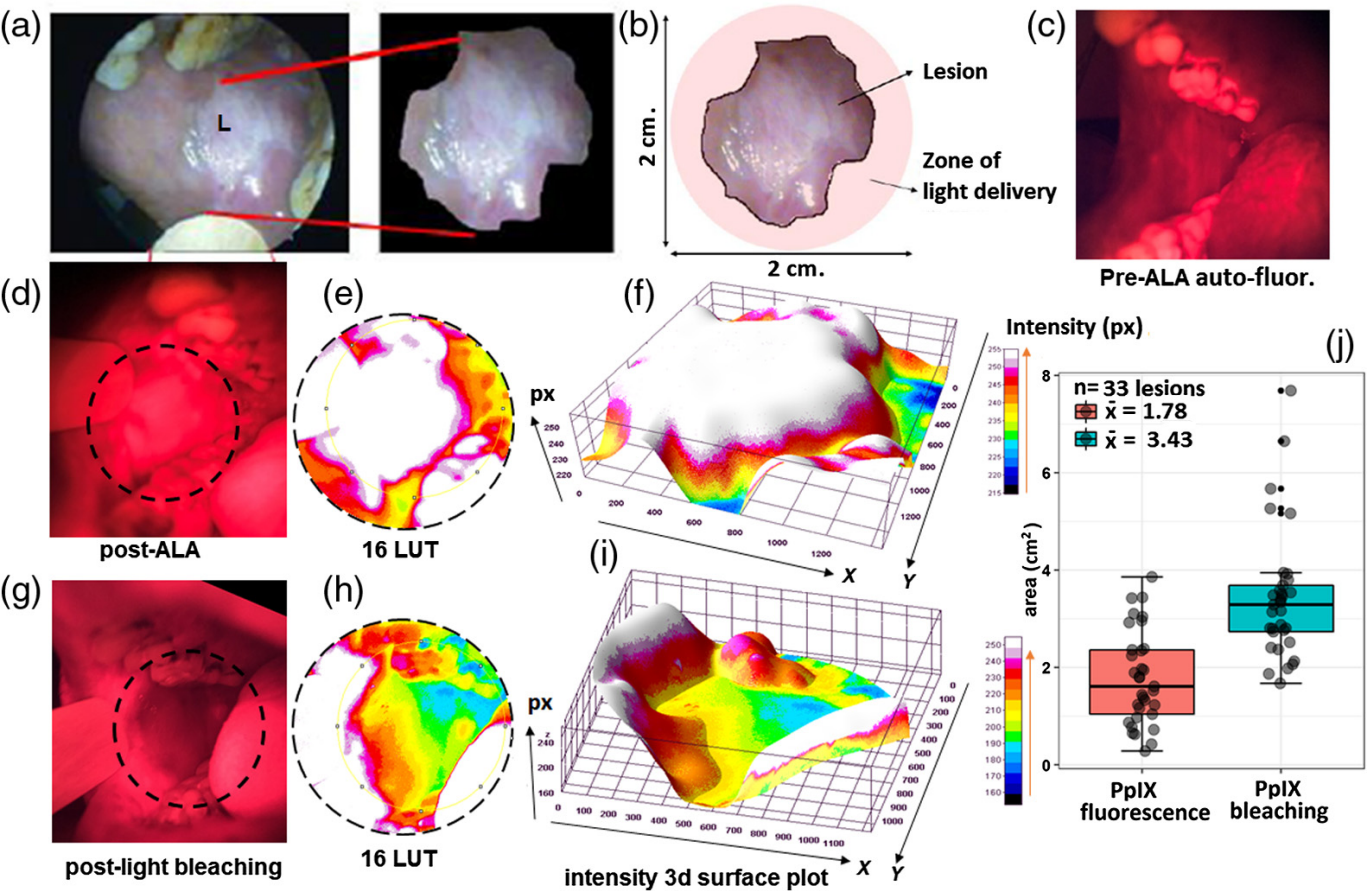
Analysis of PpIX fluorescence signal in the zone of light delivery before and after PDT. (a) and (b) Measurement of two-dimensional parameter of buccal mucosa lesion by WL imaging. (c) The pre-ALA autofluorescence imaging. (d) and (g) Pre- and post-light delivery PpIX fluorescence and bleaching imaging. (e) and (h) The corresponding lesion margin’s identification by 16LUT. (f) and (i) Lesion surfaced fluorescence intensity and bleaching were visualized by 3-D fluorescence intensity surface plot of 16LUT. (j) The comparative boxplot analysis of PpIX fluorescence and post-PDT bleaching areas. The larger area of photobleached region following PDT is consistent with expectations based on the treatment design, using a light delivery applicator, which treats the full lesion area plus margins.

Pre-ALA WL images of lesions consistently showed a characteristic shiny and semismooth white patch [Figs. 3(a) and 3(b)], whereas there is no visible tumor contrast in pre-ALA fluorescence images, which reveal approximately uniform autofluorescence background [Fig. 3(c)]. After the last dose of ALA, the lesion is visible due to the generation of PpIX fluorescence [Fig. 3(d)]. It has been reported that tumor tissue shows 12.5 times higher fluorescence signal than surrounding tissue after topical administration of ALA (200 mg) to oral lesions at 1 to 2.5 h.^26^ In our studies, after the first systemic dose of ALA (at 1 h), the PpIX fluorescence contrast begins to become visible though it is significantly enhanced after the third (final) ALA fraction [Fig. 3(d)] (see Table S1 in the Supplementary Material, for large sample size of the lesion site PpIX fluorescence). The color segmentation of red fluorescence using 16 pseudo-color LUT helps to visualize the lesion [Figs. 3(d) and 3(e)].

In images obtained after PDT light delivery, dramatic photobleaching is visible [Figs. 3(g), 3(h) and Tables S1 and S3 in the Supplementary Material)]. The observation of photobleaching is expected as some proportion of the singlet oxygen generated during PDT will react with the photosensitizer itself causing permanent photobleaching.^27^ Lesion surface fluorescence intensity and bleaching were also visualized by 3-D fluorescence intensity surface plots. The 3-D distribution of pixels intensity in 16LUT color spacing corresponds to PpIX red fluorescence and PpIX bleaching [Figs. 3(f) and 3(i)]. The photobleaching produced a 42% decrease in PpIX fluorescence at the lesion site (t=15.4; $p=2.3\times 10^{-16}$). The mean fluorescence intensity value of 133 post bleaching is close to the mean baseline autofluorescence intensity of 126 as compared to 228 in ALA-photosensitized lesions pre-PDT (Table S3 in the Supplementary Material). The photobleaching of the lesion sites is the maximum extent of the area covering the lesion site with largest mean area and smaller interquartile range ($\text{mean}=3.43\;{\mathrm{cm}}^{2}$ and $q_{3}-q_{1}=0.95\;{\mathrm{cm}}^{2}$) [Fig. 3(j), see Fig. S2 in the Supplementary Material]. In addition, the difference between the central values of PpIX fluorescence area and PpIX bleaching area variables are significantly different (t=7.55; $p=1.24\times 10^{-8}$). The significantly larger area of bleaching relative to the lesion area is consistent with treatment design, using a light delivery applicator, which provides margins of ∼5  mm beyond the edges of the target lesion [see Fig. S2 in the Supplementary Material]. The photobleaching observed is likely due to the combined effects of PpIX photobleaching (nonspecific PpIX accumulated beyond lesion boundaries) as well as bleaching of tumor and normal tissue autofluorescence. The ability to quantify the location and area of photobleaching is a useful assessment tool to confirm that light was indeed delivered to the target site. If spatially coregistered with pre-PDT imaging, the overlaid photobleaching map could also be used to confirm that light delivery achieved the desired margins around the lesion.

### 3.3 Fluorescence and White Light HSV Segmentation

**Fig. 4:**
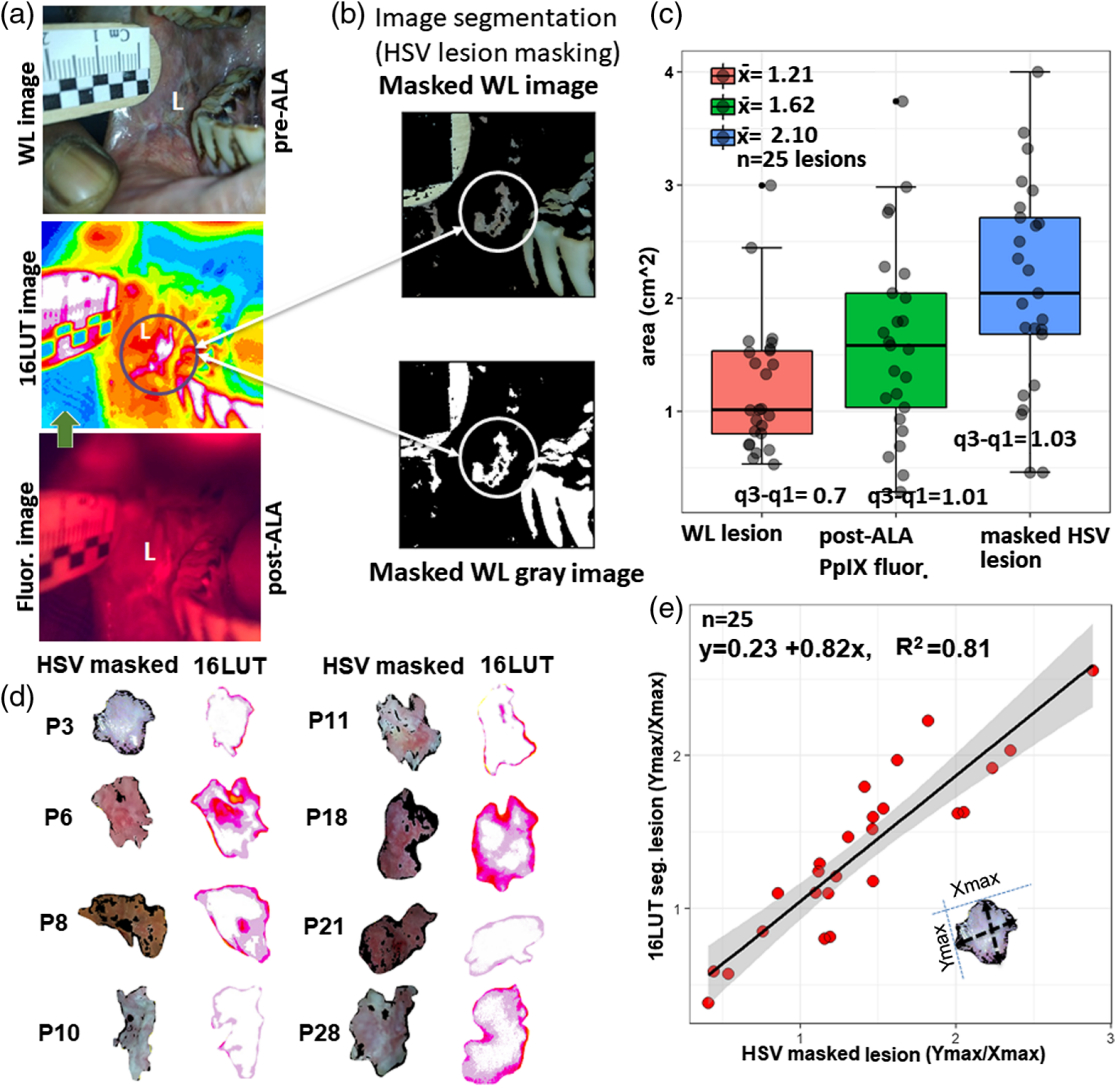
Comparative analysis of lesion segmentation based on fluorescence and WL image data. (a) The pre-ALA WL and post-ALA fluorescence imaging with corresponding lesion site 16LUT segmentation. (b) The HSV segmentations of pre-ALA WL image showing the same visible lesion dimensions as in the fluorescence image (LUT). (c) The boxplot comparison among the masked HSV, pre-ALA, WL, and post-ALA LUT lesion areas. (d) The similar visible lesion dimensions displayed by the HSV masked and LUT image segmentations. (e) The simple linear regression plot for predictor (relative axis of HSV masked lesion; axial $\text{ratio value}=Y_{\text{max}}/X_{\text{max}}$) and dependent variable (relative axis of 16LUT lesion). The shaded gray area represents the confidence interval (95%) for regression coefficients.

Although post-ALA-induced PpIX fluorescence and 16LUT contrast-enhanced images are useful to locate and demarcate the surface of lesions [Fig. 4(a)], we also sought to corroborate conclusions from fluorescence imaging by an independent analysis of lesion area based on the abnormal color and texture of the diseased tissue visible in a WL image. Here, in WL HSV color spacing segmentation, we masked the WL original image and WL gray image using threshold values of HSV [Fig. 4(b)]. Although WL images showed that the lesion size centered around $1.21\;{\mathrm{cm}}^{2}$, the post-ALA fluorescence area is larger ($\text{mean}=1.62\;{\mathrm{cm}}^{2}$) with uniform area distribution [Fig. 4(c)]. The distribution of lesion area in HSV lesions is uniform but there is a marked difference in the mean area among the PpIX fluorescence and HSV lesions (t=2.17; p=0.04). The lesion shape identified in the HSV masking is almost the same as fluorescence images [Fig. S3 and Table S2 in the Supplementary Material and Fig. 4(d)]. Furthermore, the visible lesion dimension transformed in the relative maximum axial parameters [i.e., $X_{\text{max}}/Y_{\text{max}}$; Fig. 4(e)] and identified the relationship between the pre-PDT HSV masked lesions and post-ALA PpIX 16LUT lesions. According to the linear regression analysis, $R^{2}$ is 0.81, i.e., roughly 81% of the variance found in the response or dependent variable (16LUT lesion size; relative max axis) can be explained by the predictor variable (HSV masking parameters) with $p=7.6\times 10^{-10}$. Hence, the visible lesion HSV segmentation (before ALA administration) can predict and identify the same lesion and may help in the demarcation of lesion’s outer boundary in the absence of ALA administration or as a companion to PpIX fluorescence imaging.

## 4 Conclusions

Here, we show that a simple smartphone-based attachment for PpIX fluorescence imaging is a useful tool aid for treatment guidance for intraoral PDT. Lesion areas obtained by fluorescence image data were validated by independent analysis of US images from the same sites, as well as a custom analysis based on HSV of WL images. These results indicate that the use of smartphone-based imaging can be used to inform the target area for light delivery without the need for more sophisticated medical imaging modalities, which may not be available in settings with limited medical infrastructure. It must be remembered that the fluorescence and LUT images only give an image of the surface of the lesion, whereas the US provides 3-D information. However, even in clinical sites where intraoral US imaging is available, the surface fluorescence contrast provides additional insight at the time of treatment. The analysis of fluorescence contrast also proves useful post treatment to confirm the location and diameter of the photobleached area, which has excellent contrast in the smartphone display. Here, we show in particular that the size of the photobleached region confirms margins around the lesion were achieved and size is consistent with the light applicator (and therefore beam spot size) used for treatment. The photobleaching analysis could be further leveraged for treatment monitoring, for example, by obtaining photobleaching image data during light delivery fraction breaks and interpreting feedback to inform modulation of light delivery parameters during subsequent fractions. In this study, all image analysis was performed off-line using a computer, though real-time interpretation of image data could be enabled by use of a smartphone App to enable online image segmentation and comparative analysis of pre- and post-treatment image data.^28^ The filter-based fluorescence imaging approach could be further enhanced by the use of multispectral imaging, for quantitative spectral unmixing of PpIX from autofluorescence background.^29^ Another design improvement would be to couple the phone to a handheld imaging probe with form factor similar to a commercial dental camera. This would be more conducive to imaging inside the oral cavity and likely to yield improved uniformity of illumination, resolution, and overall image quality. Nevertheless, the smartphone-based approach used here offers the advantage of requiring minimal hardware to obtain images that largely agree with US and provide essential insight about lesion size and shape to guide subsequent therapeutic light delivery.
