# Supplementary material for: Clinical evaluation of smartphone-based fluorescence imaging for guidance and monitoring of ALA-PDT treatment of early oral cancer
Source: J Biomed Opt. 2020 Apr 11;25(6):063813. doi: 10.1117/1.JBO.25.6.063813 (PMC7148420; doi:10.1117/1.JBO.25.6.063813)
Supplement: Supplementary file 1 [file JBO_025_063813_SD001.pdf]

Supplementary information

Clinical evaluation of smartphone-based fluorescence imaging for guidance and monitoring of ALA-PDT treatment of early oral cancer

Shakir Khan[1], M A Bilal Hussain[1], Amjad P Khan[2], Hui Liu[3], Shaista Siddiqui[4], Srivalleesha Mallidi[2], Paola Leon[3], Liam Daly[3], Grant Rudd[3], Filip Cuckov[3], Colin Hopper[5], Stephen Bown[5], Kafil Akhtar[6], Syed Abrar Hasan[7], Shahid Ali Siddiqui[1], Tayyaba Hasan[2*], Jonathan P. Celli[3*]

[1]Department of Radiotherapy, Jawaharlal Nehru Medical College, Aligarh Muslim University, Aligarh, India.
[2]Massachusetts General Hospital and Harvard Medical School, Boston, Massachusetts, USA.
[3]University of Massachusetts at Boston, Boston, Massachusetts, USA.
[4]Department of Radiodiagnosis, Jawaharlal Nehru Medical College, Aligarh Muslim University, Aligarh, India.
[5]University College London, London.
[6]Department of Pathology, Jawaharlal Nehru Medical College, Aligarh Muslim University, Aligarh, India
[7]Department of Oto-Rhino-Laryngology (E.N.T.), Jawaharlal Nehru Medical College, Aligarh Muslim University, Aligarh, India.

*Address all correspondence to Prof. Tayyaba Hasan, E-mail: thasan@mgh.harvard.edu and Dr. Jonathan Celli; E-mail: Jonathan.Celli@umb.edu

Table of contents	Page no.
**Supplementary Figure S1:** The scatterplot between lesion area and lesion width measured in LUT of PpIX fluorescence images.	2
**Supplementary Figure S2:** The boxplot of pre-PDT white light (WL) lesion area, post-ALA PpIX induced fluorescence area of the lesions and post light treatment bleaching area.	3
**Supplementary Figure S3:** (a) The pre-PDT lesion site HSV masking and post-ALA lesion site PpIX fluorescence in 16LUT. (b) The bar-graph of the relative maximum axis of the lesions identified by HSV masking and 16LUT image segmentation.	4
**Supplementary Table S1:** Lesion's site PpIX fluorescence and bleaching image segmentation.	5
**Supplementary Table S2:** Lesion's WL-HSV image segmentation and correlation with fluorescence imaging.	10
**Supplementary Table S3:** Lesion's site auto-fluorescence, PpIX fluorescence and PpIX bleaching images.	15

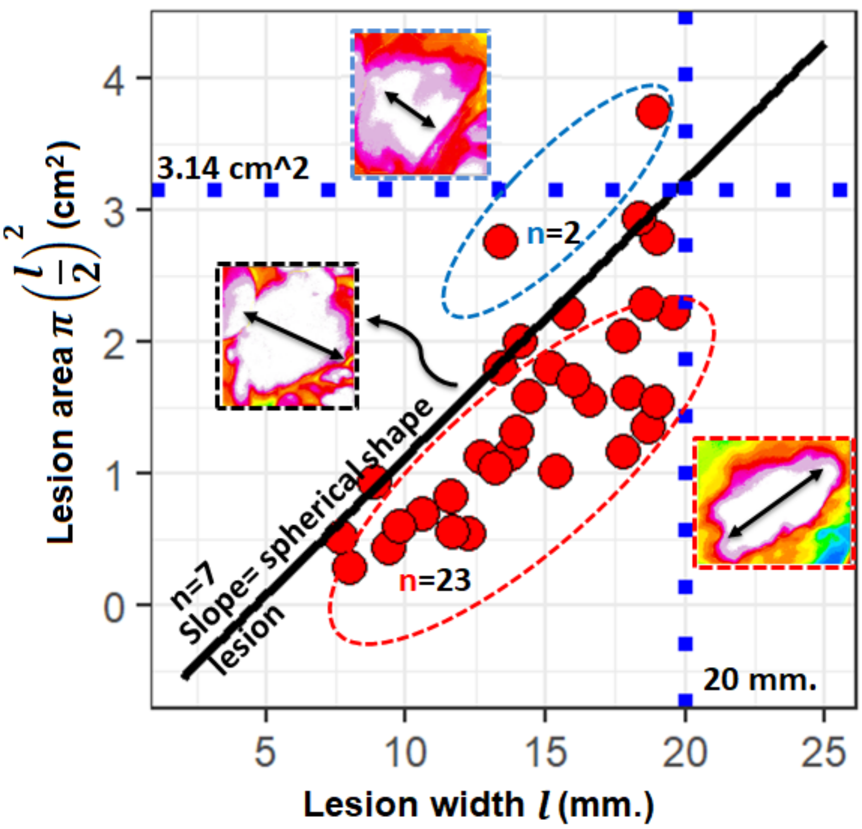

Supplementary Figure S1: The scatterplot between lesion area and lesion width measured in LUT of PpIX fluorescence images. The black line slope represents the calibrated perfectly spherical shape of lesions where 7 lesions fall upon the line and showing the nearly spherical shape morphology. Under the line 23 lesions showing the elongated shape lesions.

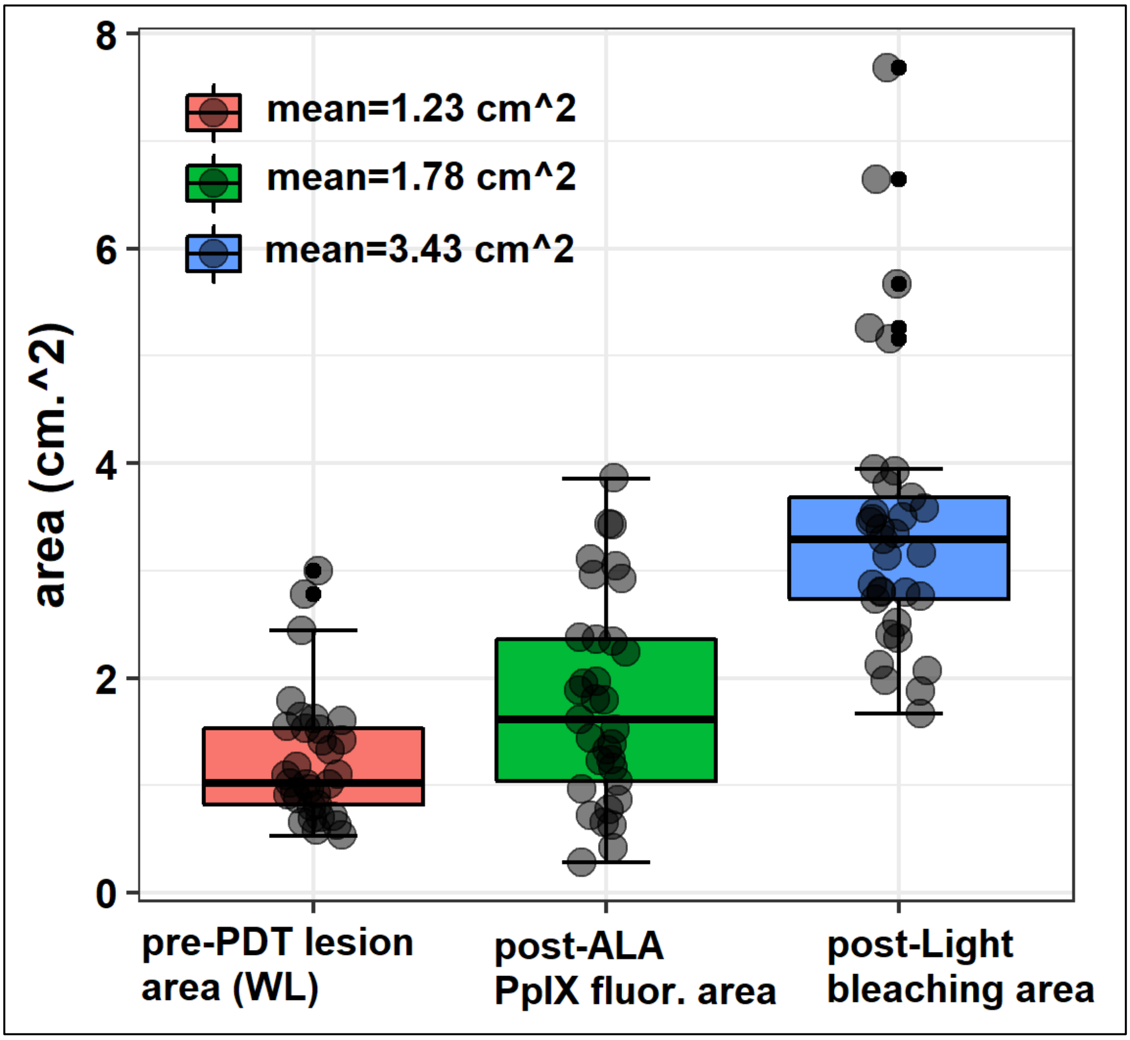

Supplementary Figure S2: The boxplot of pre-PDT white light (WL) lesion area, post-ALA PpIX induced fluorescence area of the lesions and post light treatment bleaching area. (n=33 lesions).

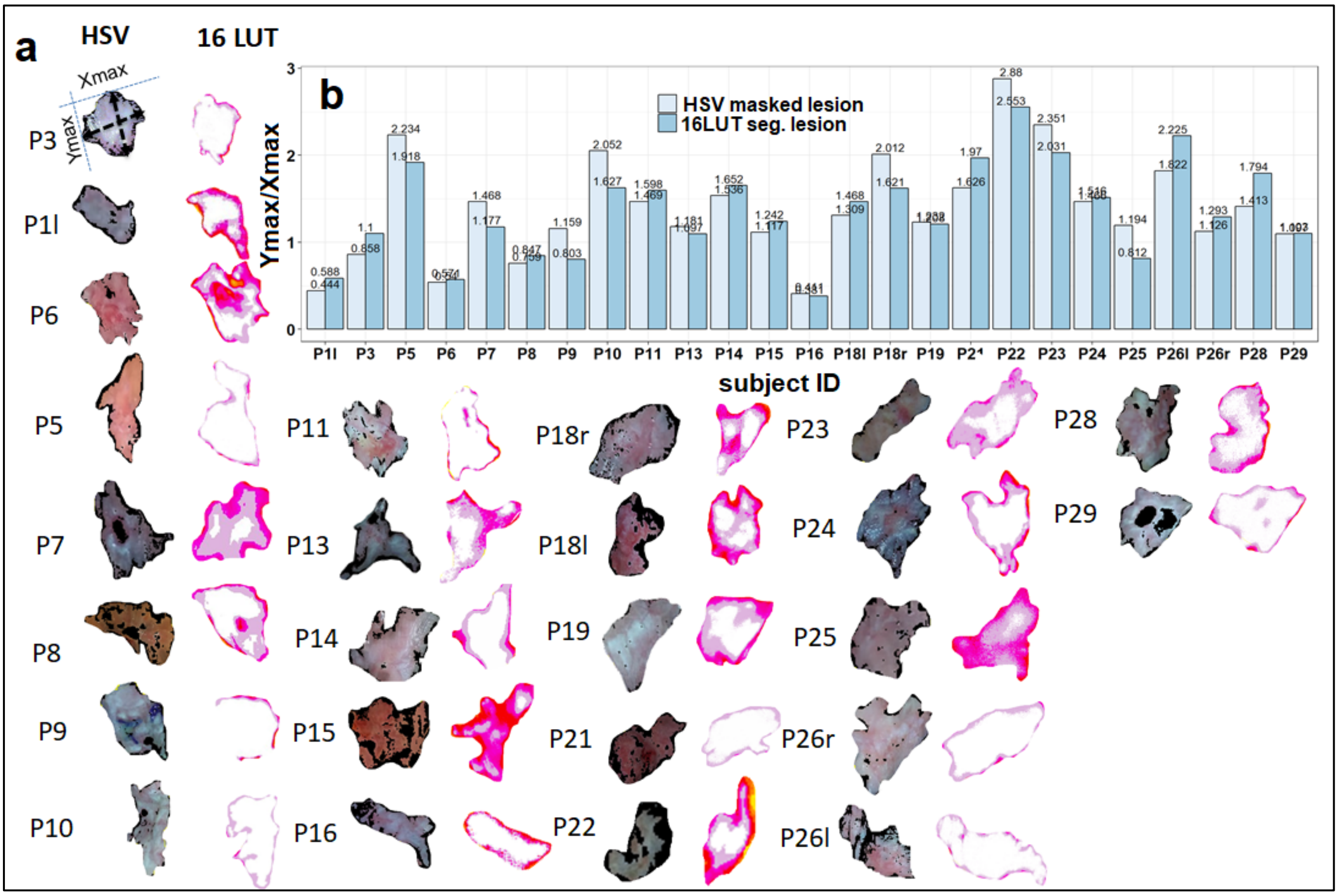

Supplementary Figure S3: (a) The pre-PDT lesion site HSV masking and post-ALA lesion site PpIX fluorescence in 16LUT. (b) The bar-graph of the relative maximum axis of the lesions identified by HSV masking and 16LUT image segmentation. (n=25 lesions).

S. Table S1: Lesion's site PpIX fluorescence and bleaching image segmentation.

**Sub. ID**	**White light lesion**	**PpIX fluorescence**	**16LUT (PpIX fluorescence)**	**PpIX bleaching**	**16LUT (PpIX bleaching)**
P11	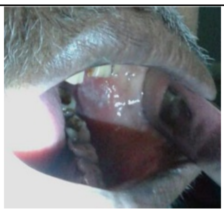	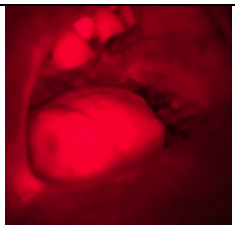	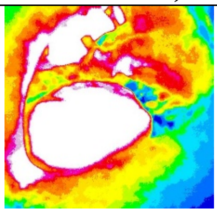	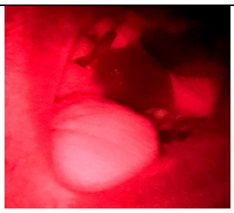	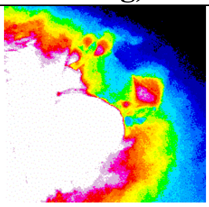
P2	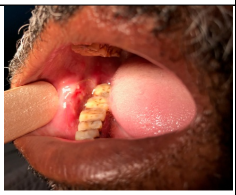	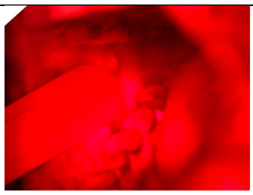	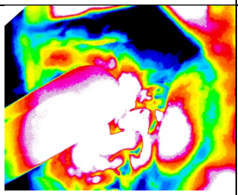	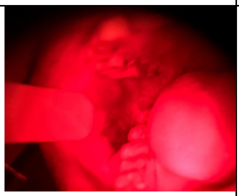	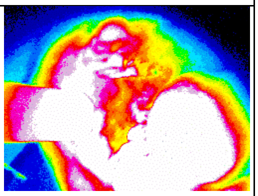
P3	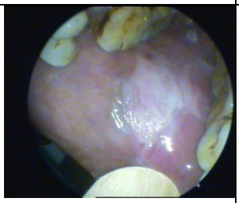	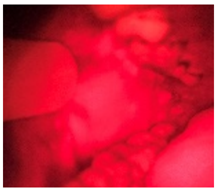	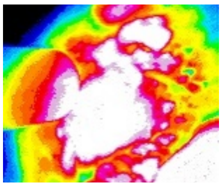	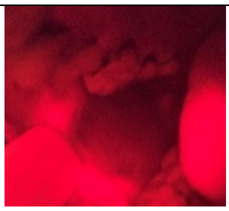	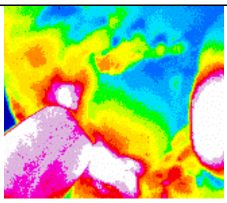
P4	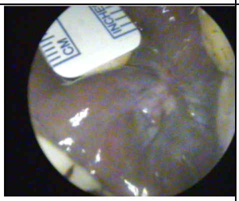	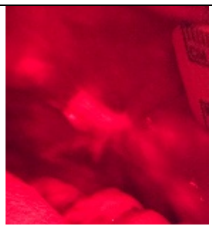	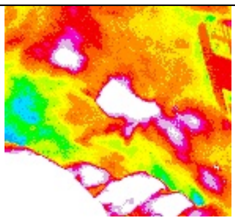	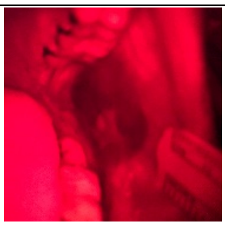	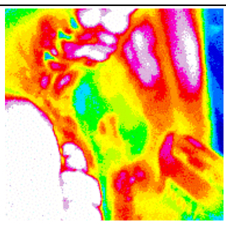
P5	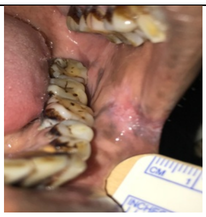	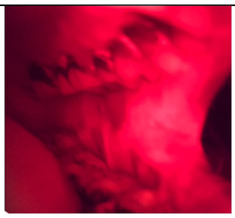	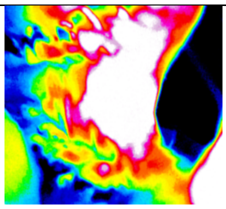	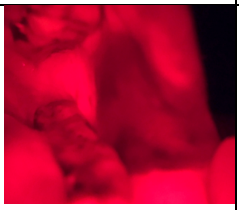	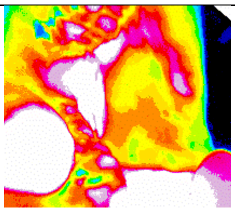
P6	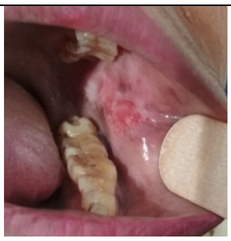	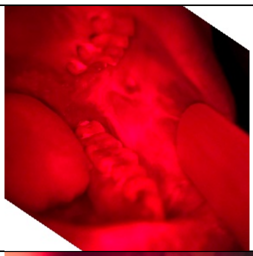	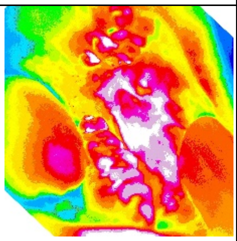	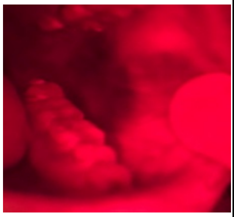	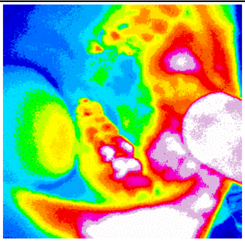
P7	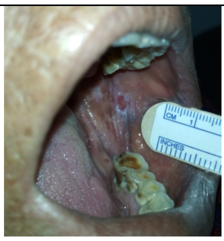	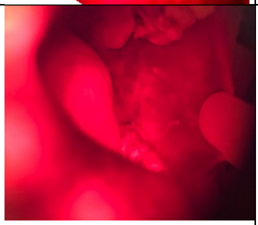	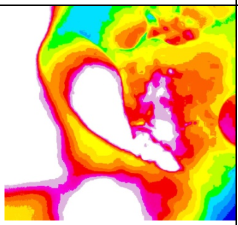	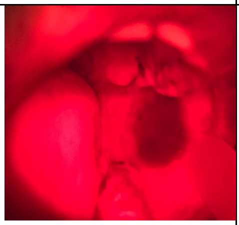	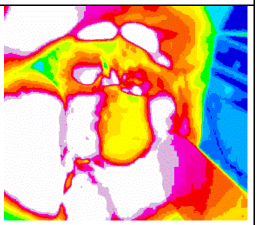

Sub. ID	White light lesion	PpIX fluorescence	16LUT (PpIX fluorescence)	PpIX bleaching	16LUT (PpIX bleaching)
P8	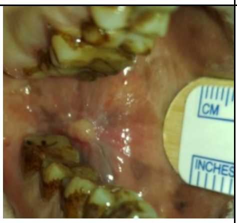	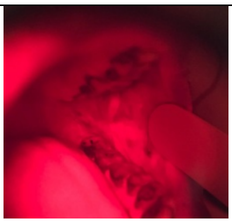	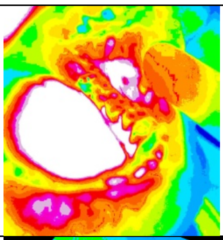	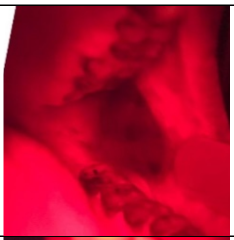	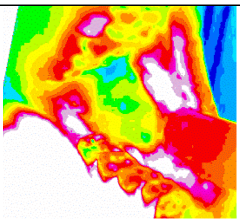
P9	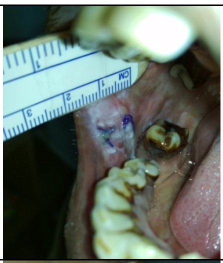	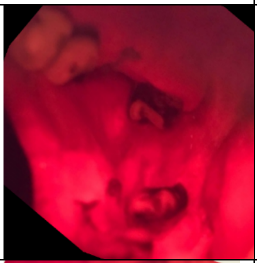	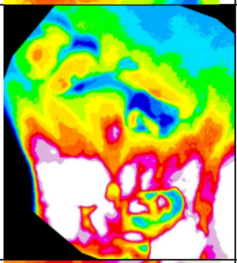	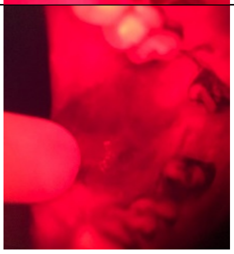	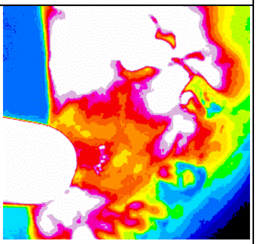
P10	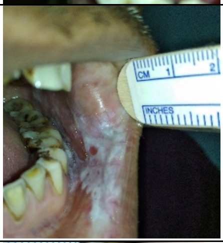	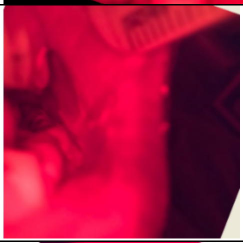	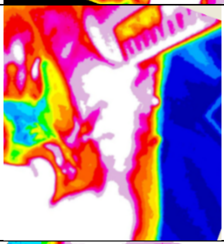	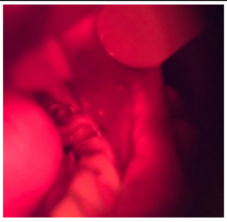	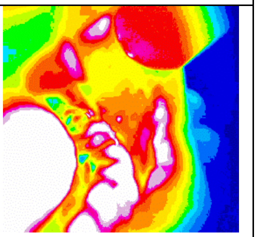
P11	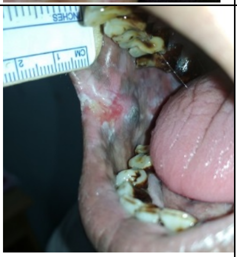	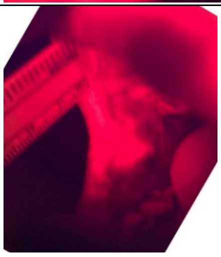	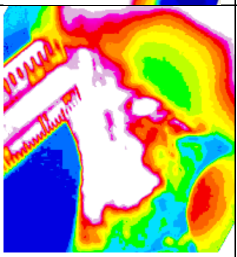	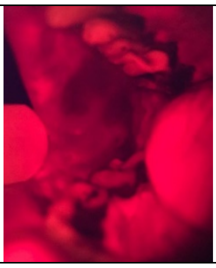	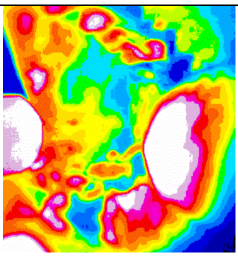
P13	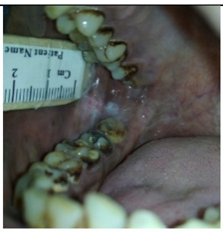	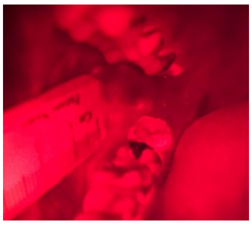	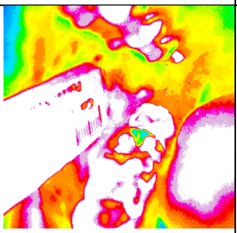	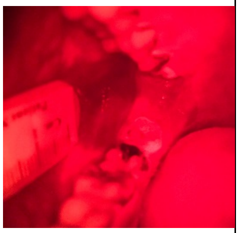	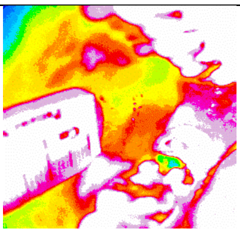
P14	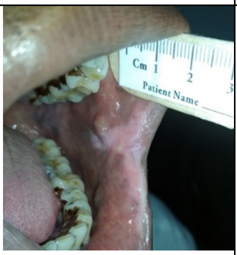	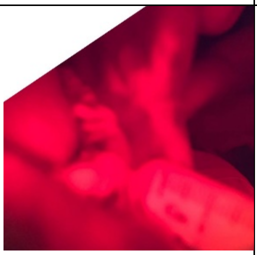	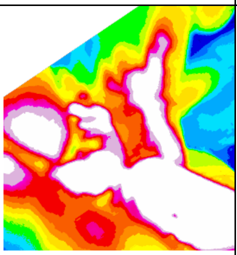	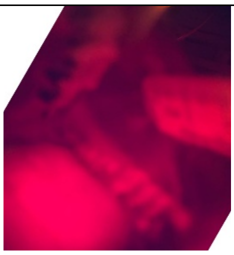	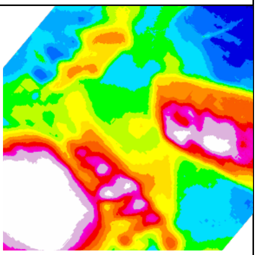

Sub. ID	White light lesion	PpIX fluorescence	16LUT (PpIX fluorescence)	PpIX bleaching	16LUT (PpIX bleaching)
P15	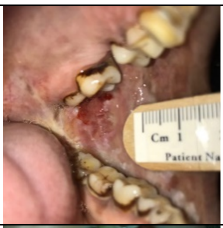	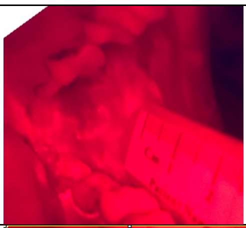	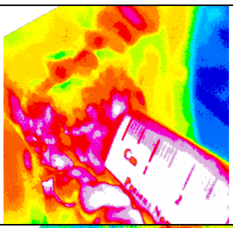	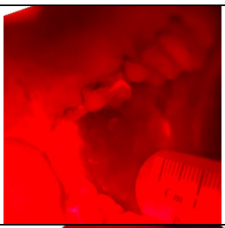	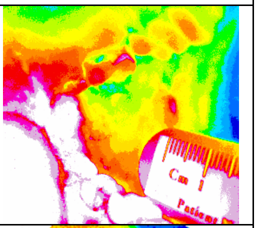
P16	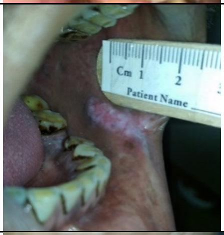	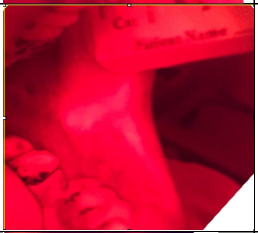	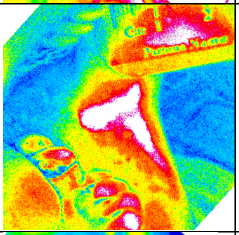	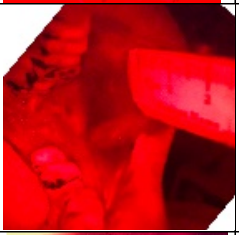	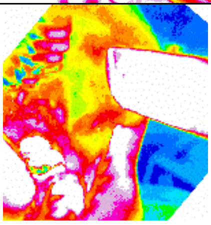
P18l	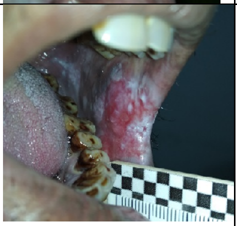	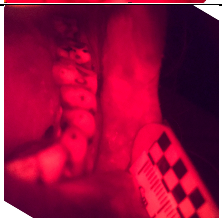	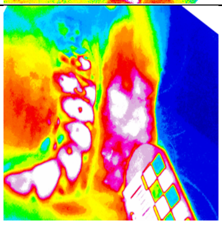	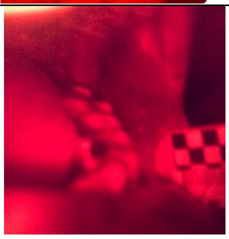	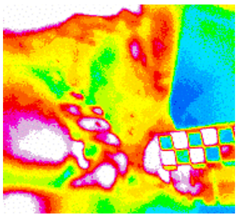
P18r	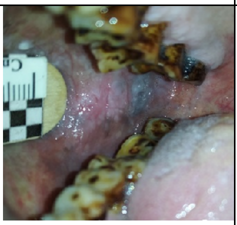	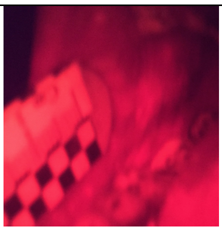	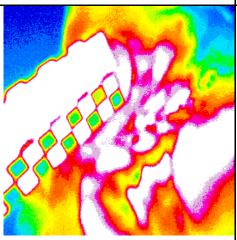	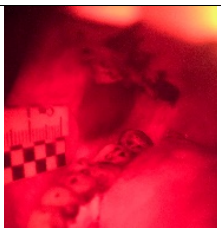	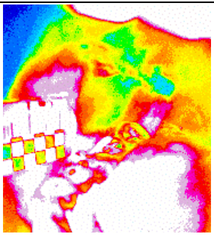
P19	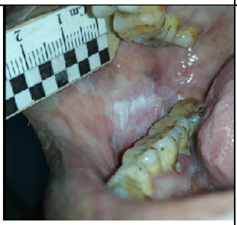	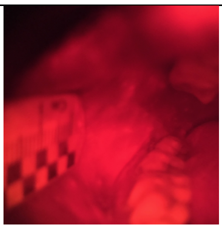	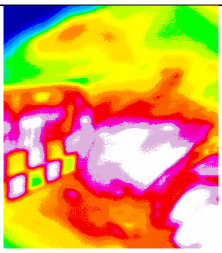	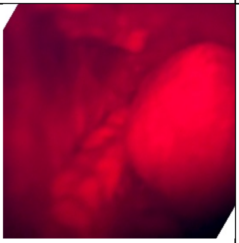	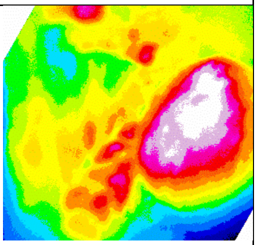
P21	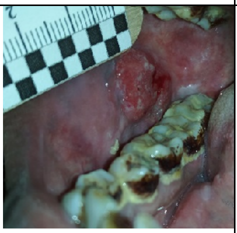	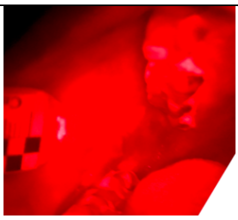	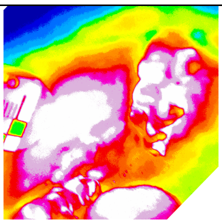	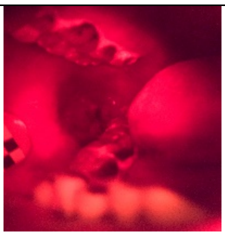	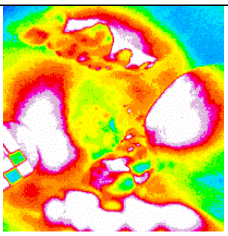

Sub. ID	White light lesion	PpIX fluorescence	16LUT (PpIX fluorescence)	PpIX bleaching	16LUT (PpIX bleaching)
P22	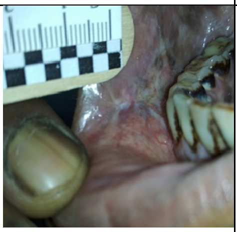	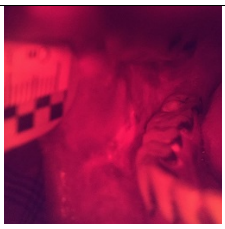	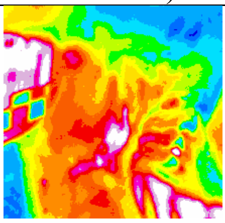	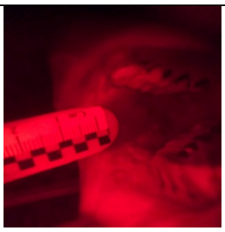	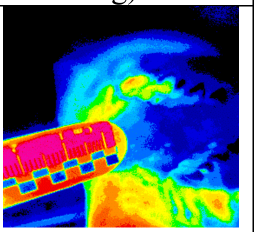
P23	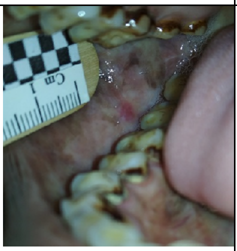	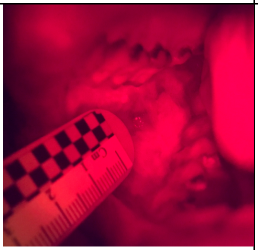	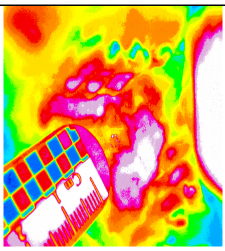	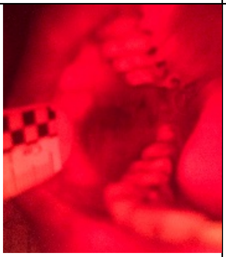	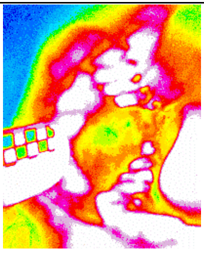
P24	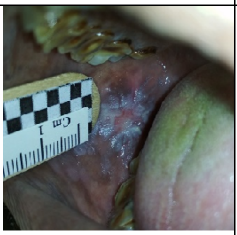	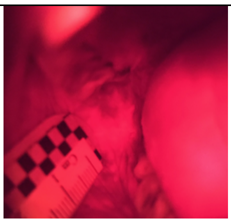	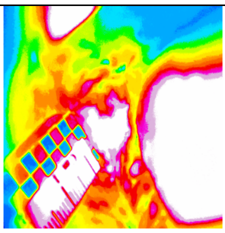	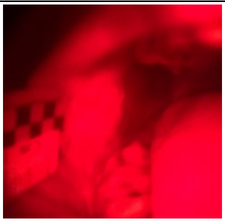	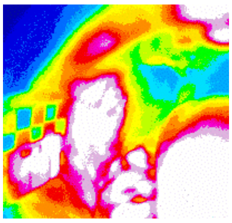
P25	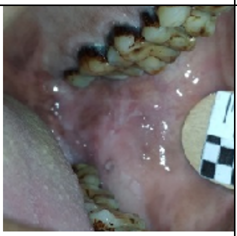	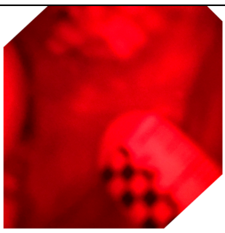	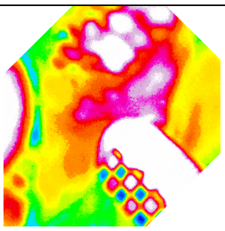	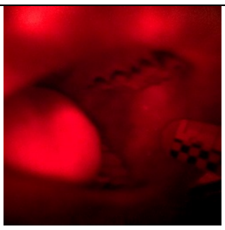	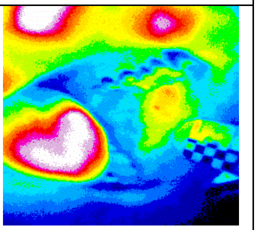
P26r	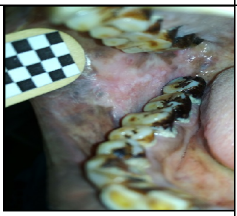	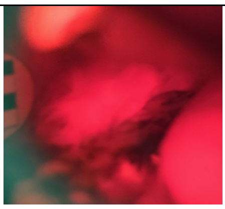	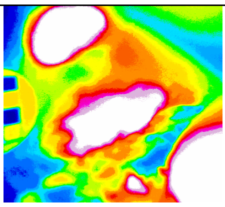	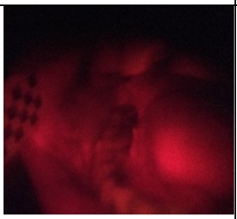	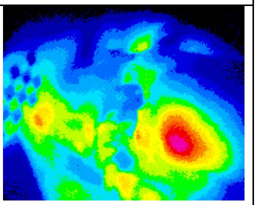
P27	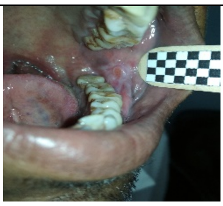	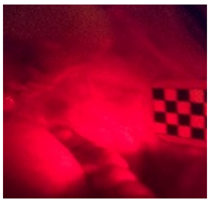	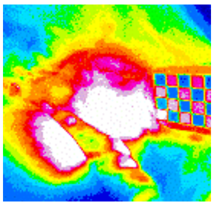	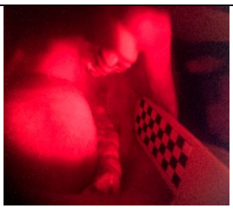	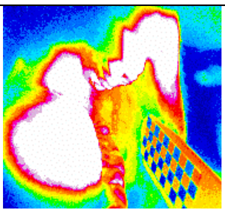

Sub. ID	White light lesion	PpIX fluorescence	16LUT (PpIX fluorescence)	PpIX bleaching	16LUT (PpIX bleaching)
P29	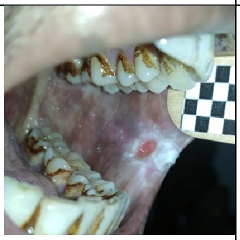	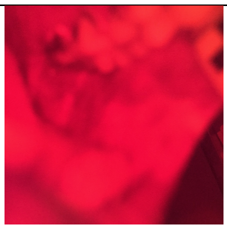	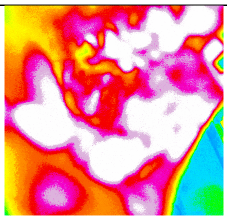	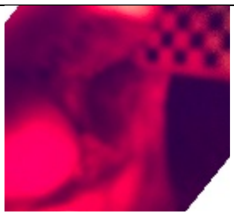	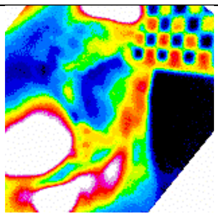

Supplementary Table S2: Lesion's WL-HSV image segmentation and correlation with fluorescence imaging.

Sub. ID	Original image	Fluorescence image	16LUT image	Masked wl (original) -HSV image	Masked wl (gray)-HSV image
P11	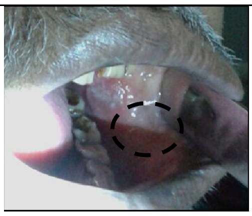	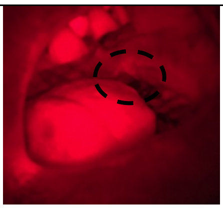	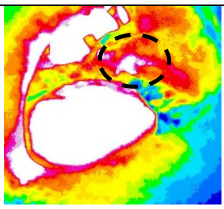	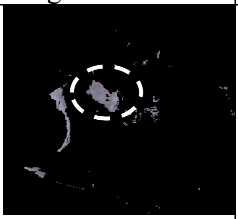	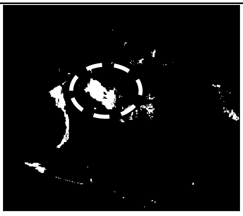
P3	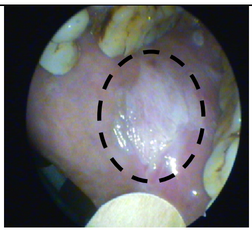	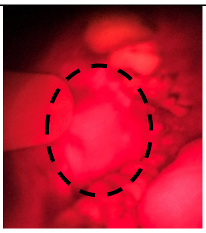	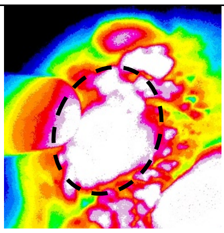	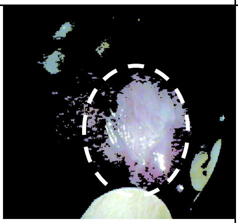	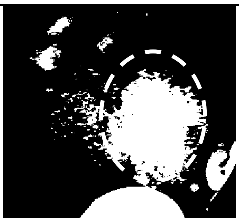
P5	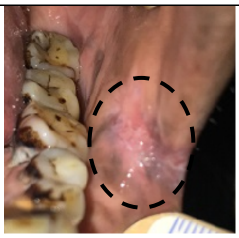	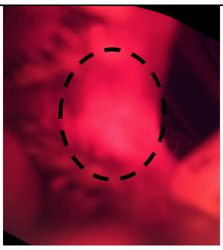	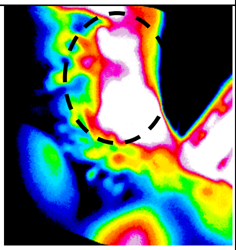	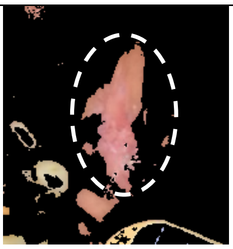	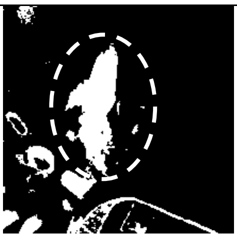
P6	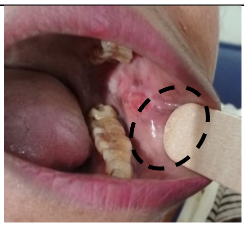	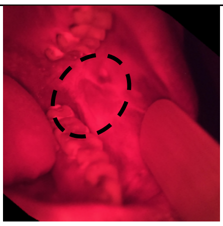	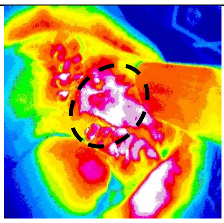	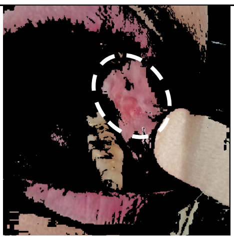	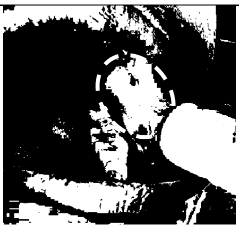
P7	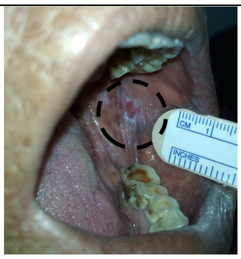	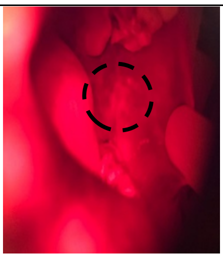	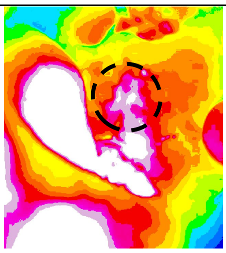	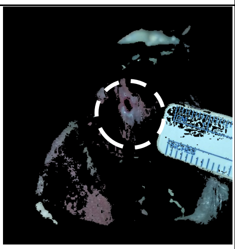	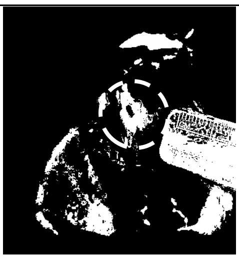
P8	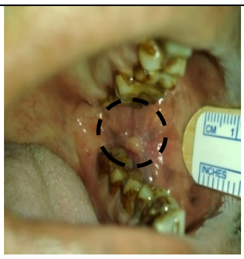	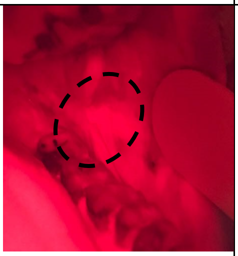	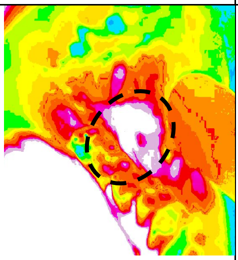	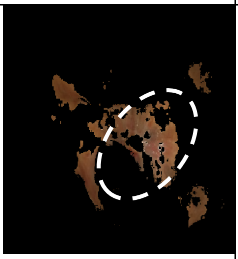	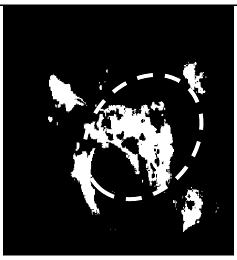

Pt ID	Original image	Fluorescence image	16LUT image	Masked wl (original) -HSV image	Masked wl (gray)-HSV image
P9	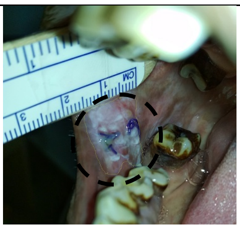	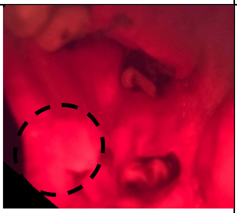	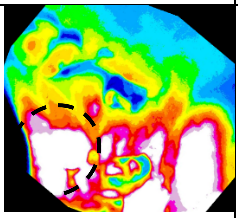	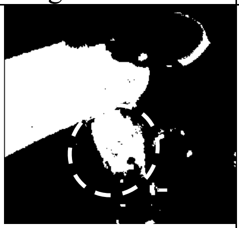	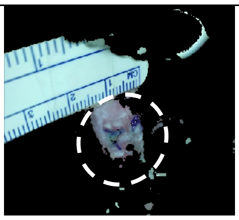
P10	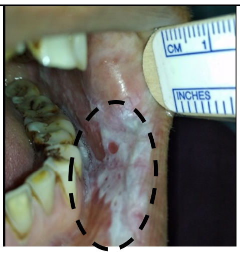	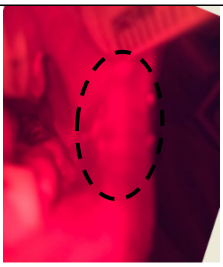	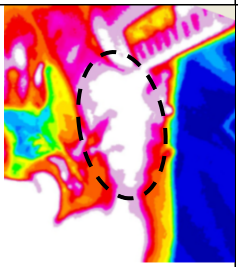	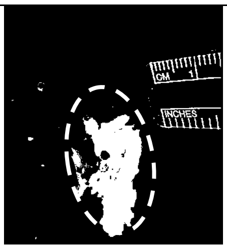	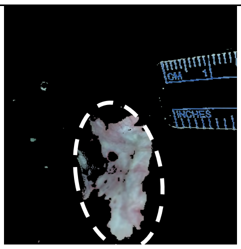
P11	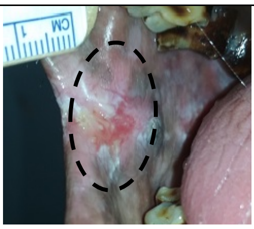	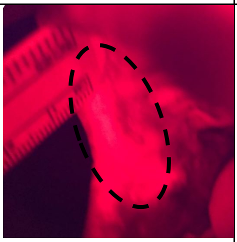	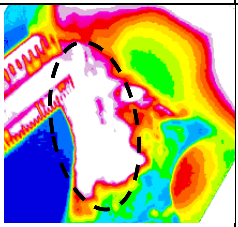	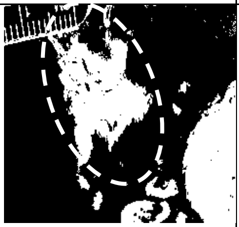	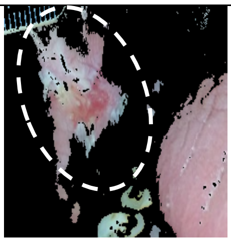
P13	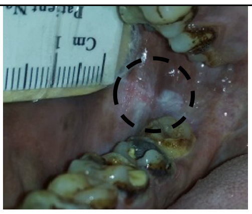	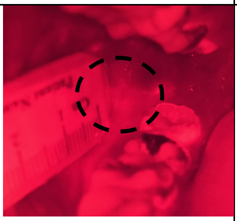	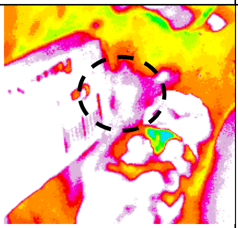	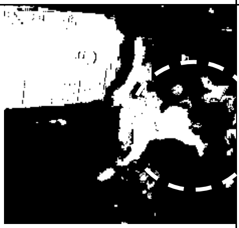	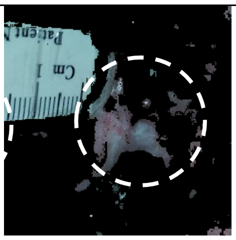
P14	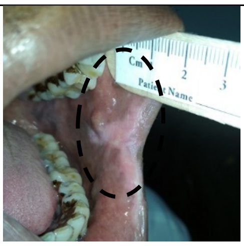	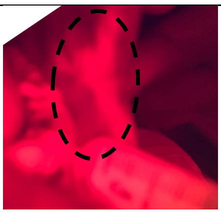	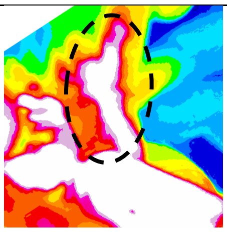	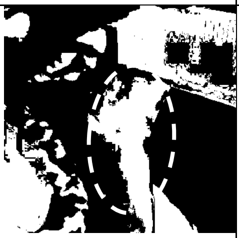	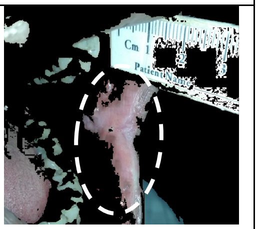
P15	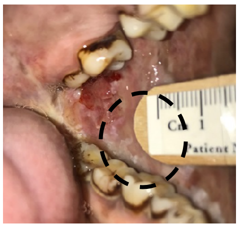	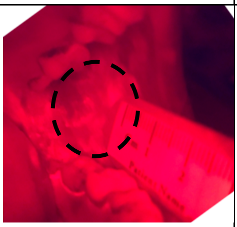	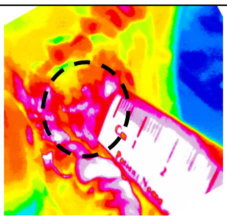	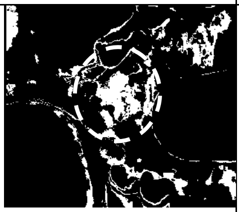	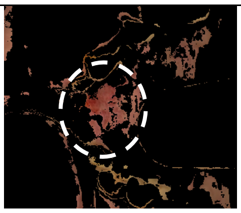

Pt ID	Original image	Fluorescence image	16LUT image	Masked wl (original) -HSV image	Masked wl (gray)-HSV image
P16	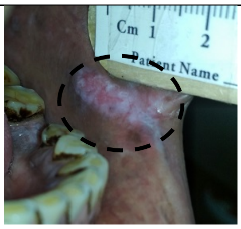	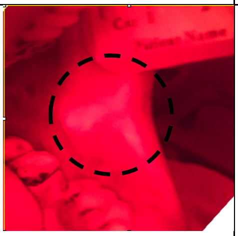	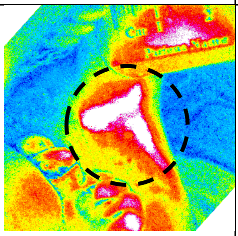	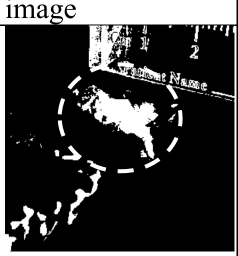	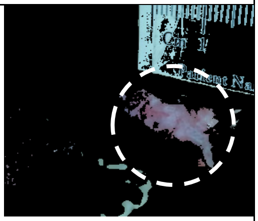
P18l	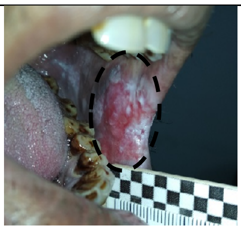	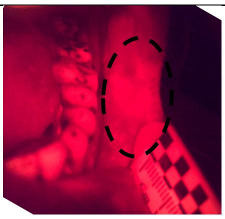	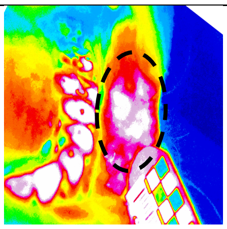	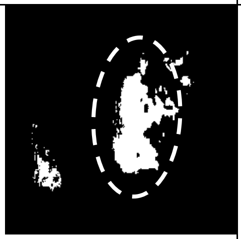	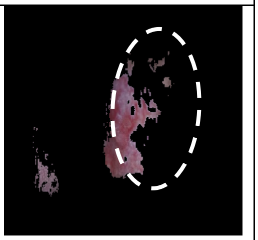
P18r	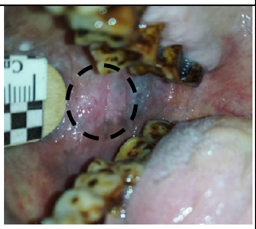	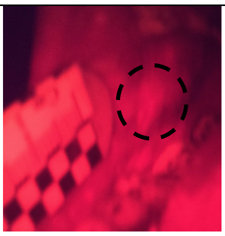	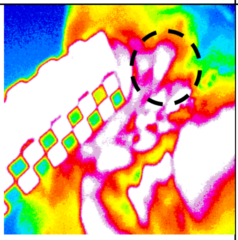	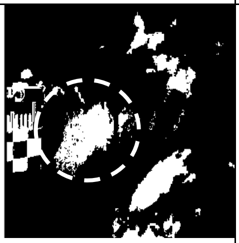	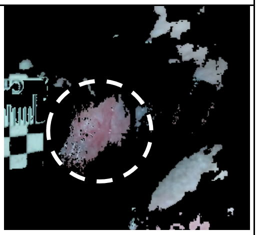
P19	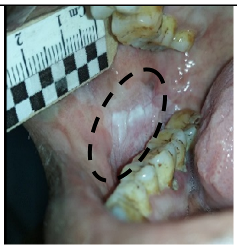	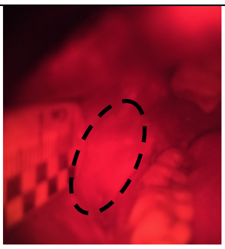	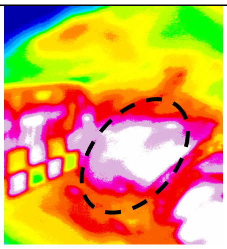	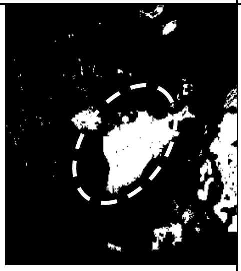	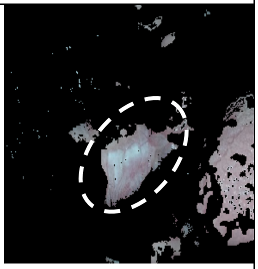
P21	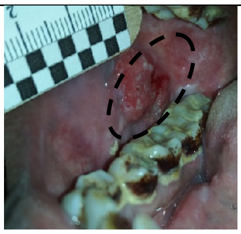	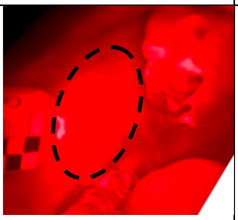	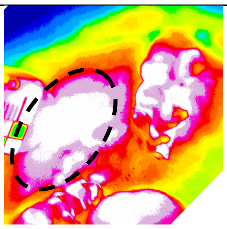	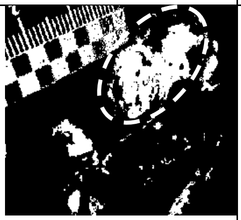	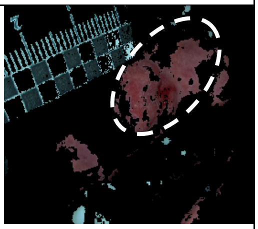
P22	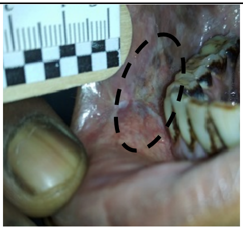	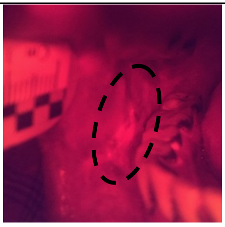	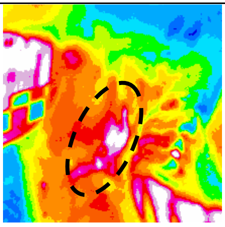	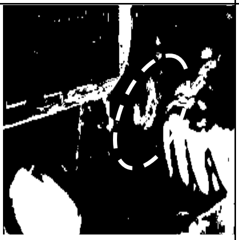	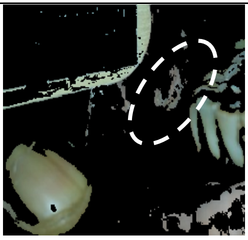

Pt ID	Original image	Fluorescence image	16LUT image	Masked wl (original) -HSV image	Masked wl (gray)-HSV image
P23	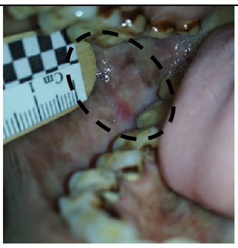	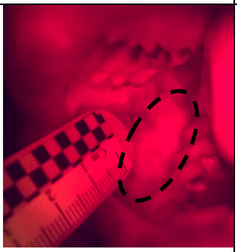	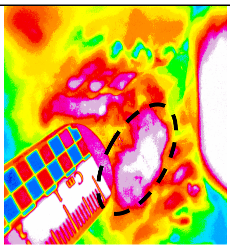	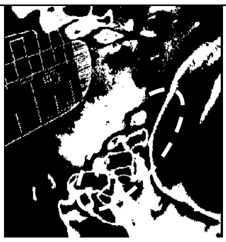	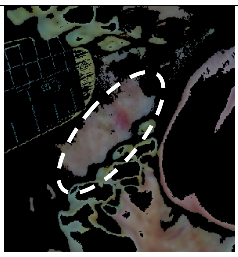
P24	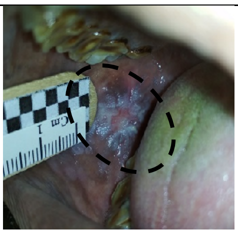	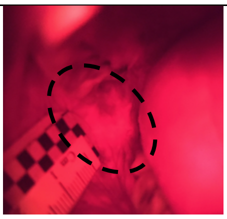	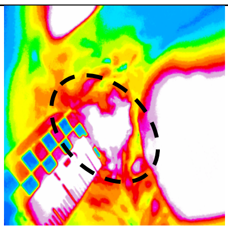	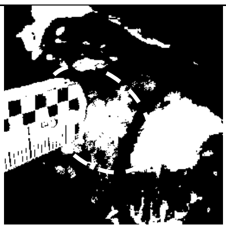	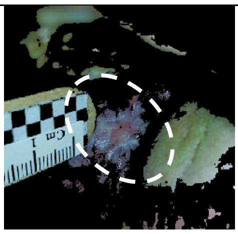
P25	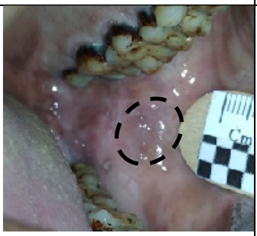	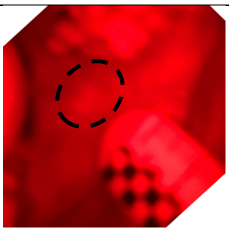	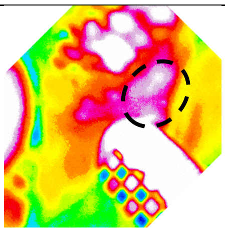	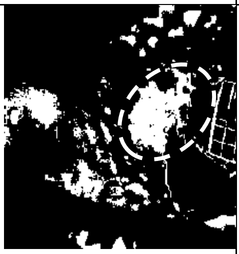	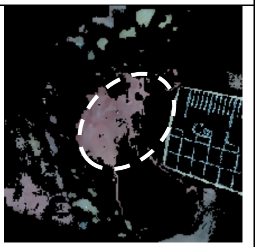
P26l	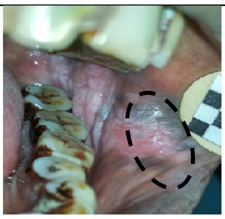	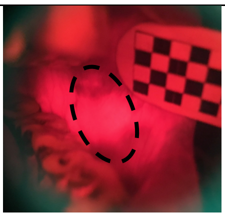	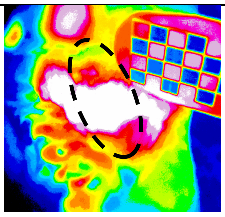	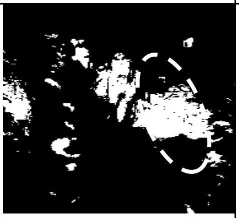	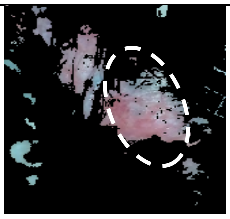
P26r	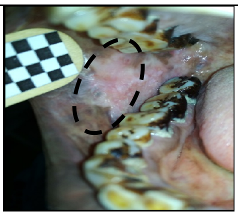	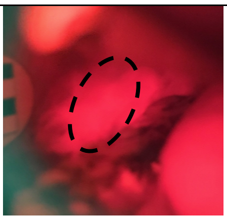	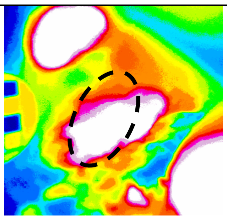	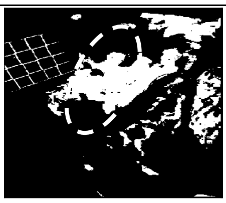	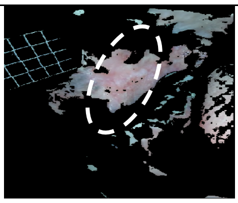
P28	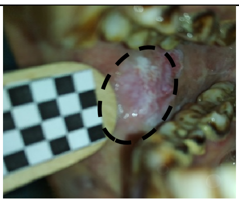	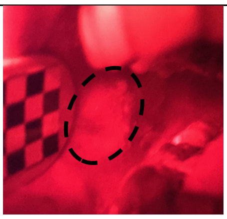	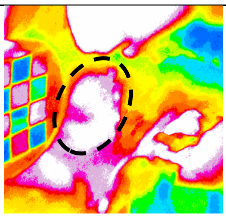	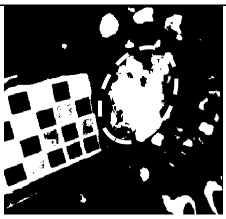	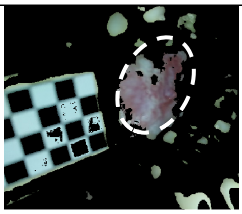

Pt ID	Original image	Fluorescence image	16LUT image	Masked wl (original) -HSV image	Masked wl (gray)-HSV image
P29	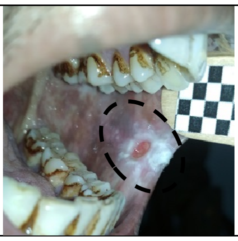	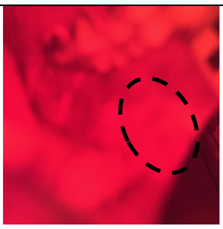	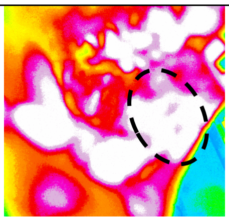	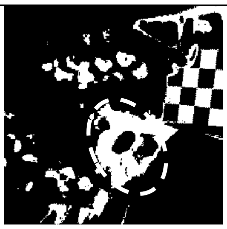	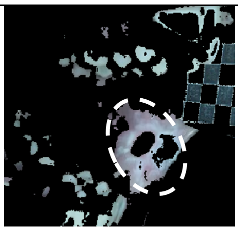

S. Table S3: Lesion's site auto-fluorescence, PpIX - fluorescence and PpIX bleaching images.

**Sub. ID**	**Auto-fluorescence**	**PpIX fluorescence**	**PpIX bleaching**
P2	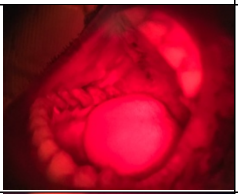	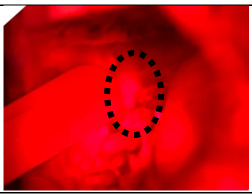	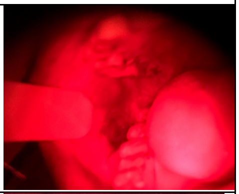
P3	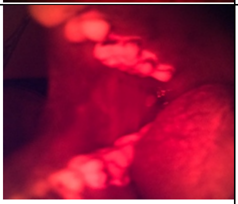	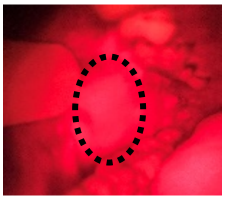	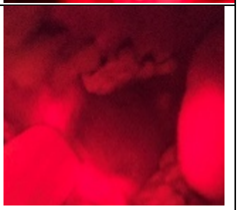
P4	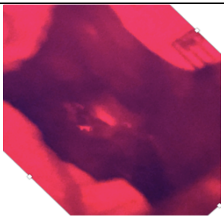	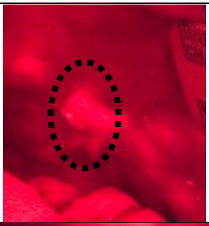	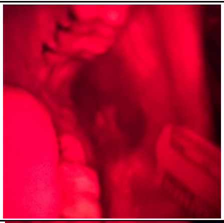
P5	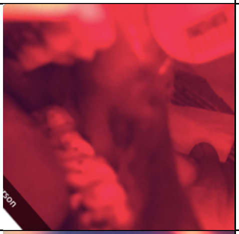	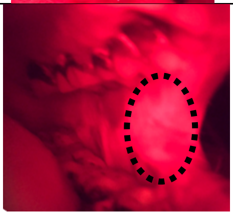	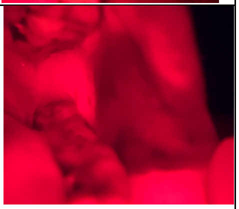
P6	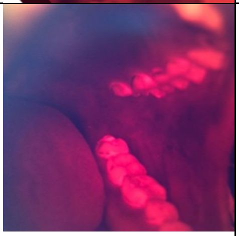	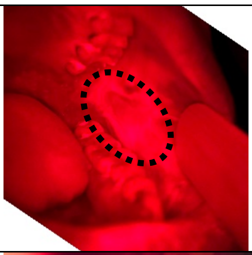	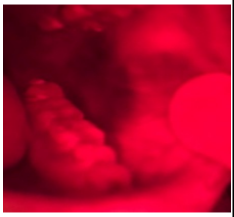
P7	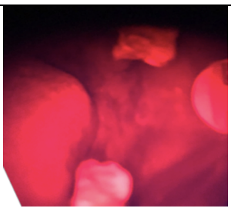	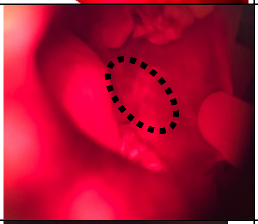	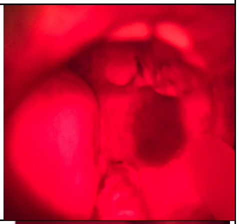
P8	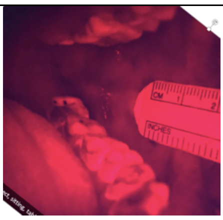	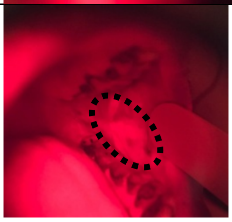	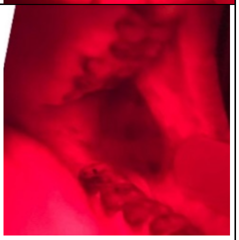

Sub. ID	Auto-fluorescence	PpIX fluorescence	PpIX bleaching
P9	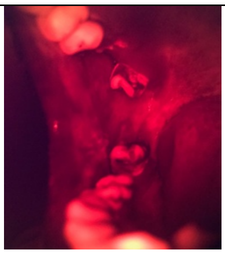	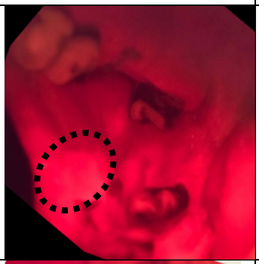	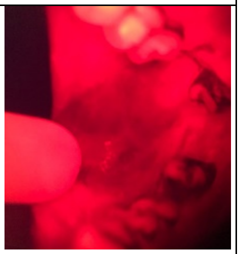
P10	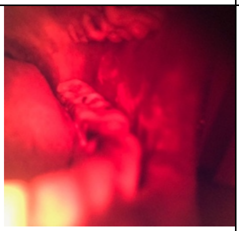	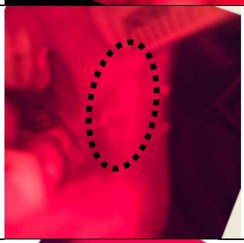	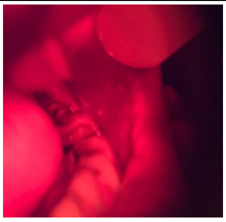
P11	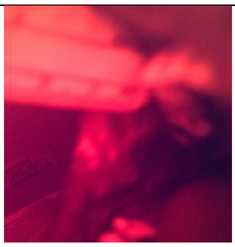	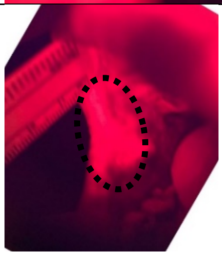	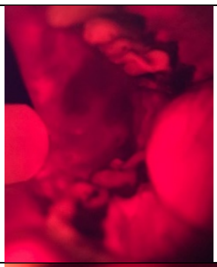
P12	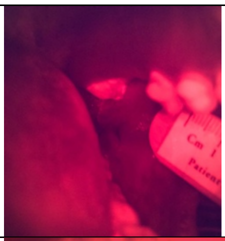	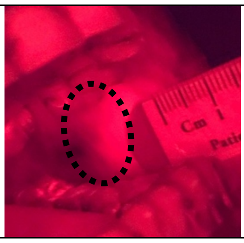	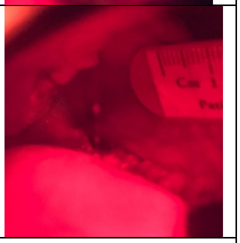
P13	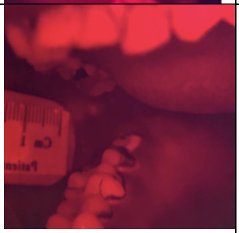	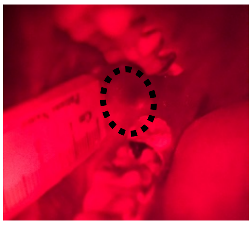	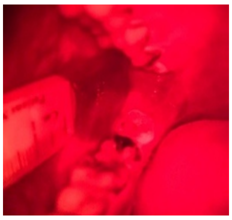
P14	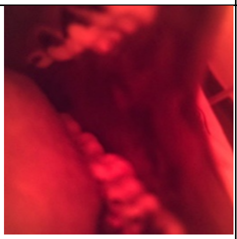	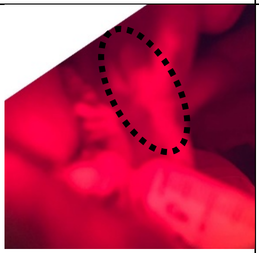	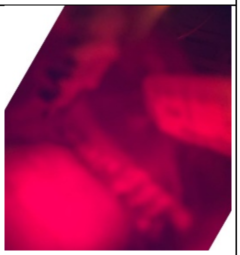

Sub. ID	Auto-fluorescence	PpIX fluorescence	PpIX bleaching
P15	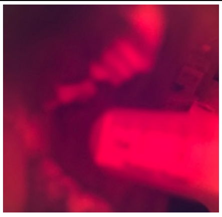	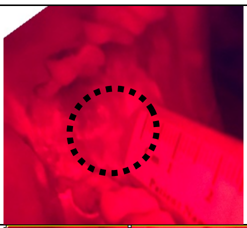	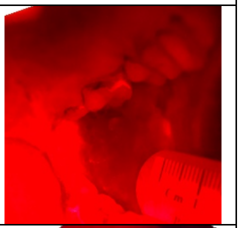
P16	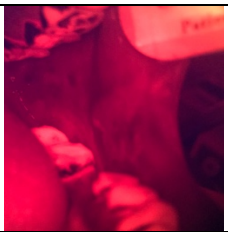	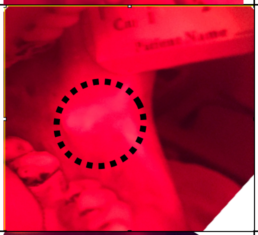	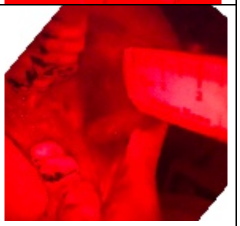
P18l	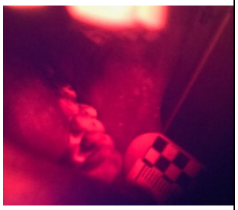	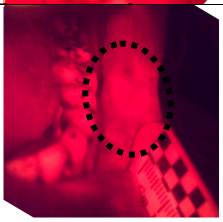	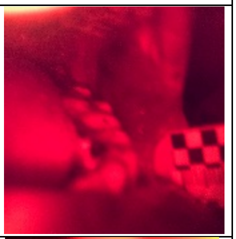
P18r	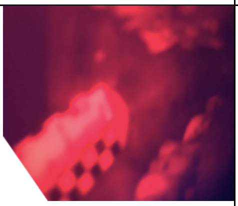	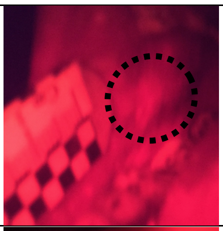	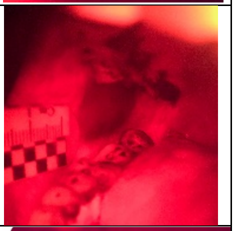
P19	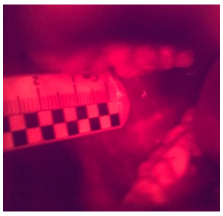	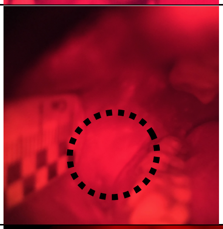	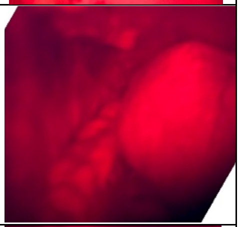
P21	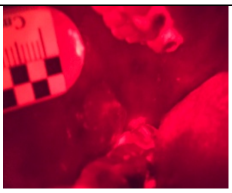	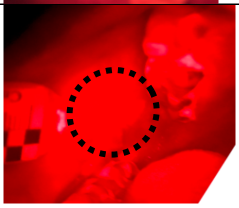	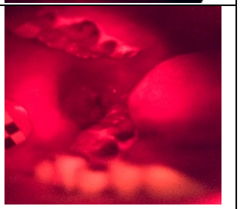
P22	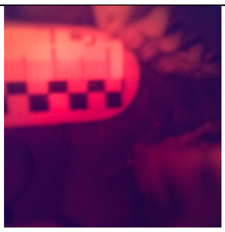	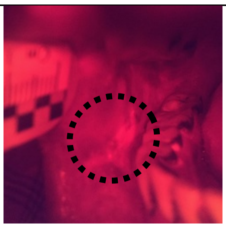	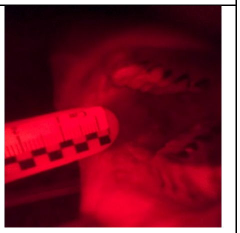
**Sub. ID**	**Auto-fluorescence**	**PpIX fluorescence**	**PpIX bleaching**

P23	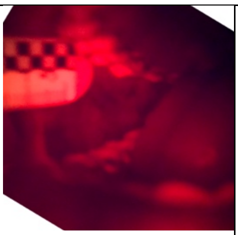	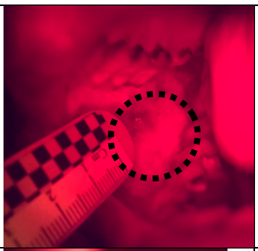	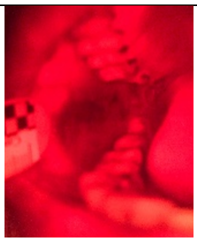
P24	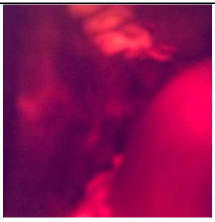	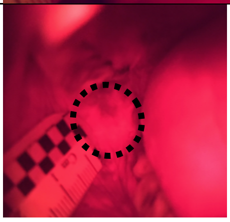	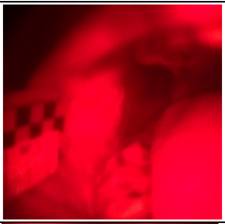
P25	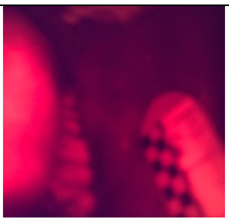	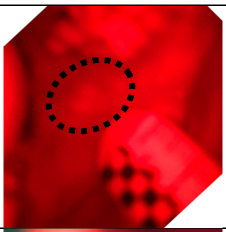	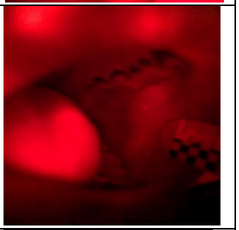
P26r	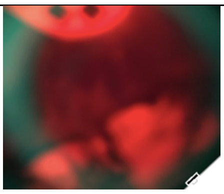	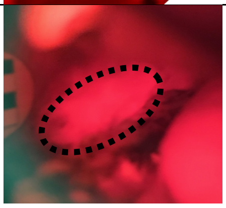	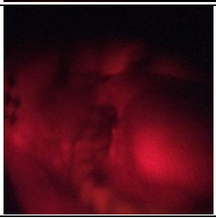
P26l	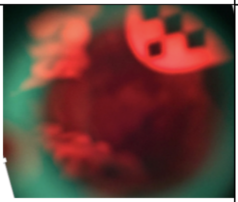	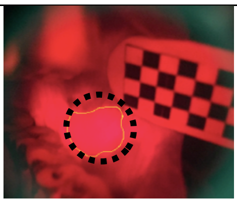	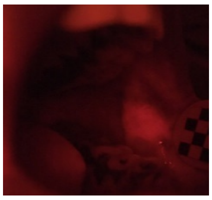
P27	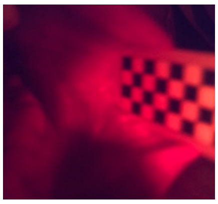	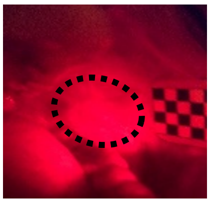	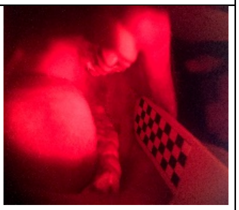
P29	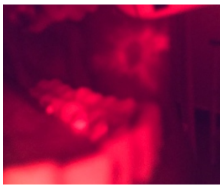	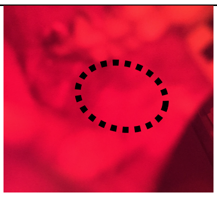	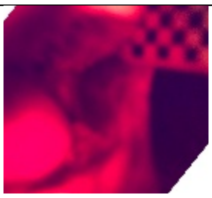
